# Tumacrophage: macrophages transformed into tumor stem-like cells by virulent genetic material from tumor cells

**DOI:** 10.18632/oncotarget.19320

**Published:** 2017-07-18

**Authors:** Yizhuang Zhang, Na Zhou, Xiuyan Yu, Xuehui Zhang, Shanxin Li, Zhen Lei, Ruobi Hu, Hui Li, Yiqing Mao, Xi Wang, Jinshu Zhang, Yuan Li, Hongyan Guo, David M. Irwin, Gang Niu, Huanran Tan

**Affiliations:** ^1^ Department of Pharmacology, Peking University, Beijing, China; ^2^ Department of Clinical Laboratory, The 305 Hospital of People’s Liberation Army, Beijing, China; ^3^ Department of Gynaecology and Obstetrics, Peking University Third Hospital, Beijing, China; ^4^ Department of Laboratory Medicine and Pathobiology, University of Toronto, Toronto, Canada; ^5^ N & N Genetech Company, Ltd., Beijing, China

**Keywords:** macrophages, circulating tumor cells, apoptosis, phagocytosis, epithelial tumors

## Abstract

Tumor-associated macrophages are regarded as tumor-enhancers as they have key roles in the subversion of adaptive immunity and in inflammatory circuits that promote tumor progression. Here, we show that cancer cells can subvert macrophages yielding cells that have gained pro-tumor functions. When macrophages isolated from mice or humans are co-cultured with dead cancer cell line cells, induced to undergo apoptosis to mimic chemotherapy, up-regulation of pro-tumor gene expression was identified. Phagocytosis of apoptotic cancer cells by macrophages resulted in their transformation into tumor stem (initiating)-like cells, as indicated by the expression of epithelial markers (e.g., cytokeratin) and stem cell markers (e.g., Oct4) and their capability to differentiate *in vitro* and self-renew in serum-free media. Moreover, we identified a subset of monocytes/macrophages cells in the blood of cancer (breast, ovarian and colorectal) patients undergoing chemotherapy that harbor tumor transcripts. Our findings uncover a new role for macrophages in tumor development, where they can be transformed into tumor-like cells, potentially by horizontal gene transfer of tumor-derived genes, thus, by taking advantage of chemotherapy, these transformed macrophages promote tumor metastasis by escaping immune surveillance.

## INTRODUCTION

Recalcitrant tumors are attributed to recurrence and metastasis [1] and the discoveries of cancer stem (initiating) cells (CSCs) and circulating tumor cells (CTCs) explain these processes and identified new targets for treating cancer [2, 3]. Cancer stem cells exist as circulating tumor cells, thus are able to seed tumors at new sites and explain the origin of metastasis [4, 5]. The origin of CSCs has long been controversial. The heterogeneity of cancer, however, has made it difficult to study CSC and CTC subpopulations directly. To date, stem cells for leukemia are the best described [6–8], where it has been shown that through a series of mutations that accumulate during somatic growth mutation-overloaded cells develop into malignant stem cells [9].

Various types of evidence challenge this model for the origins of other types of cancer and it is likely that a single explanation cannot explain all cancers [10, 11]. It has been suggested that other types of cells must be capable of being drivers to replenish tumor cell populations [12–14]. During development of a tumor within a body, tumor cells constantly interact with other types of cells, such as mononuclear phagocytes, a component of the immune system, which play important roles in tumor surveillance and regulation of the cellular microenvironment [9, 15–18]. Cells from the monocyte-macrophage lineage have long been recognized as heterogeneous [19]. Since lineage-defined subsets of these cells have not been identified, macrophage heterogeneity probably reflects the plasticity and versatility of these cells to respond to micro-environmental signals.

Macrophages are a major component of the leukocyte infiltrate of tumors [18, 20, 21]. Activation and polarization, in response to stimulation by cytokines and microbial agents, yield two main states for macrophages: classically activated M1 and alternatively activated M2 types [19]. M1 macrophages are potent effector cells that kill microorganisms and tumor cells and can produce copious amounts of pro-inflammatory cytokines [19, 22]. M2 cells tune the inflammatory response and adaptive Th1 immunity, scavenge debris, and promote angiogenesis, tissue remodeling and repair [18]. Evidence suggests that some M2 type macrophages participate in inflammatory circuits that promote tumor progression [20, 22]. Scavenger receptors, such as scavenger receptor A, scavenger receptor B and CD163, are typically expressed at higher levels by M2 compared to M1 macrophages [23–27].

Previous work has shown that macrophages, which phagocytize dead cells, can acquire DNA from apoptotic bodies [28]. Cells undergoing apoptosis, a process that frequently occurs to cancer cells, generate apoptotic bodies. Therefore, macrophages, through phagocytosis, potentially can acquire genetic material from cancer cells [29–31], and thus tumor-associated macrophages (TAMs) might not only acquiring pro-tumor DNA but also pro-tumor factions that may transform them into tumor cells with advanced malignant behaviors [29–33]. In view of the diverse roles and fates of macrophages, we investigated the consequences of the interaction of macrophages with apoptotic tumor cells: (i) Would interactions with tumor cells potentiate pro-tumor functions in macrophages? (ii) What changes occur after tumor DNA is phagocytized by macrophages? Would macrophages be transformed into tumor cells, or even tumor stem cells, by horizontal gene transfer? (iii) Do tumor-like monocyte/macrophages, which might promote tumor progression, exist in the circulation of cancer patients?

## RESULTS

### Monocytes from patients with different types of epithelial tumors are double positive for CD163 and EpCAM

**Figure 1:**
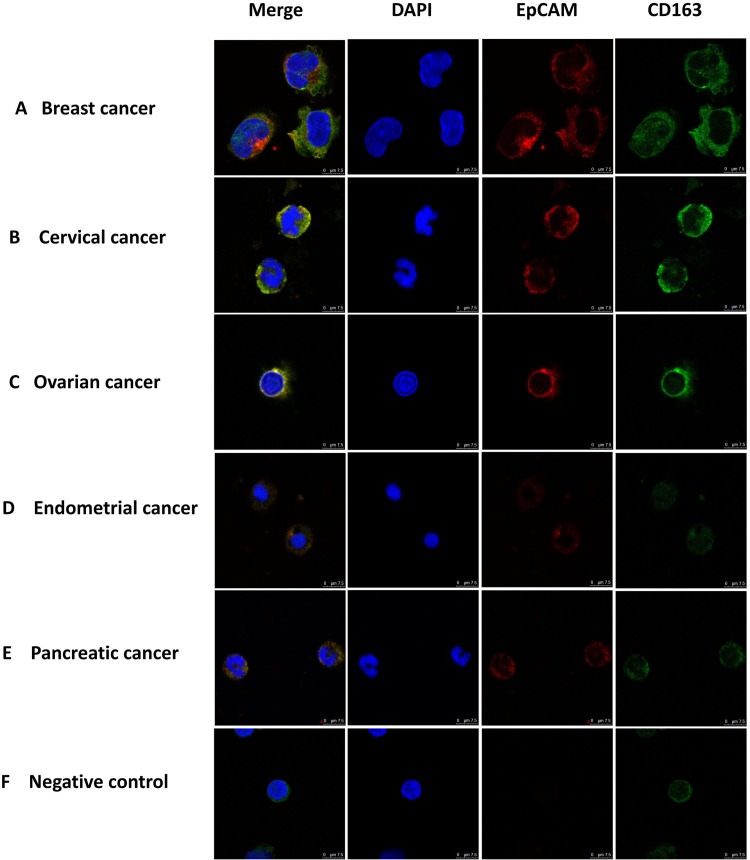
Immuno-staining of monocytes from patients with different types of epithelial cancers Monocytes were stained with antibodies to CD163 (green) and EpCAM (red), with nuclie stained with DAPI (blue) and imaged under a confocal microscope. Merged image is shown on the left. Monocytes were enriched from the blood of patients with different types of epithelial tumors using Ficoll Paque-plus density gradient centrifugation. Monocytes were from a patient with breast **(A)**, cervical **(B)**, ovarian **(C)**, endometrial **(D)**, and pancreatic **(E)** cancer as well as a healthy individual **(F)** negative control. Scale bar, 75μm.

As a first step in identifying and characterizing CTCs in cancer patients we examined blood cells using immunofluorescence with antibodies against EpCAM, a widely used biomarker for cancers of the epithelial lineage [14, 34–37]. EpCAM positive cells were readily identified in blood samples from patients with cancers derived from several distinct epithelial sources (Figure 1). To distinguish CTCs from macrophages we used antibodies against CD163, which belongs to the scavenger receptor family and are a marker for M2 macrophages [23–25]. Surprisingly, we found cells that displayed both CD163 and EpCAM immunofluorescence in blood from patients with a variety of types of patients (Figure 1A-1E), including breast, cervical, ovarian, endometrial and pancreatic cancer, but not in healthy subjects (Figure 1F). Images, at lower magnification, showing increased numbers of EpCAM and CD163 double-positive monocyte cells are presented in Supplementary Figure 1. These observations suggest that monocyte-derived macrophages in patients with epithelial cell-derived cancers might have interacted with CTCs and acquired biomarkers from these cells.

### Phagocytosis of apoptotic cancer cells by mouse peritoneal macrophages

Apoptosis and phagocytosis potentially explain the acquisition of cancer biomarkers by macrophage cells seen in Figure 1. Apoptosis frequently occurs in adult life, especially in tumor tissues where the proliferation of cells is dysregulated and in a nutrient restricted environment [38, 39]. Many factors, including cancer therapy (if given), outgrowth of nutrient supply and host immune actions can initiate apoptosis in tumor cells [39, 40]. Macrophages are a major immunocyte population in tumors, accounting for 60% of the infiltrating immune system cells [18], and through phagocytosis clear the cellular debris generated by apoptosis [29, 41–43]. To test whether cancer biomarkers could be transferred from cancer cells to macrophage cells we established an *in vitro* apoptosis-phagocytosis model, where purified macrophages were co-cultured with cells that had been induced to experience apoptosis. Viable cells were removed from our model, to prevent them from influencing downstream results. The MCF-7 cancer cell line was used as the primary model for our experiments. Apoptosis was induced in the MCF-7 cancer cell line using hydrogen peroxide (H_2_O_2_), as previously done by others [44], achieving apoptosis in 100% of the MCF-7 cells with a concentration of 0.3mM at a cell concentration of 5×10^6^ or 10×10^6^ (Supplementary Figure 2A-2C). For all our following experiments, we used 0.3mM H_2_O_2_ to induce apoptosis in 5×10^6^ MCF-7 cells.

Mouse peritoneal macrophages were isolated and co-cultured with human MCF-7 cells, or primary mouse hepatocytes that had not received H_2_O_2_ treatment as a control, for our co-culture model. Very low levels of apoptosis were detected by flow cytometry in control cells (Supplementary Figure 2D). To follow interactions between mouse macrophages and MCF-7 (tumor) or hepatocyte (control) cells, CellTracker was used to differentially label the cells. A green probe was used to label the mouse macrophages while a red label was used for the MCF-7 or hepatocyte cells. After labeling with CellTracker, macrophages were co-cultured with MCF-7 or hepatocyte cells for 3h and then examined under a fluorescence microscope. Only a very small number of cells contained both red and green labels when untreated cells were examined (Supplementary Figure 2E-2F), indicating that little phagocytosis occurred between macrophages and live MCF-7 or hepatocyte cells.

To determine whether apoptosis increased the rate of phagocytosis, apoptosis was induced in labeled MCF-7 cells, and the harvested dead cells and apoptotic bodies incubated with mouse peritoneal macrophages at ratios of 1:1 and 2:1 (MCF-7 cells:macrophages) for 3h. After co-culture, cells were analyzed by CD11b/PI flow cytometry (Supplementary Figure 2G-2H) and immunofluorescence (Supplementary Figure 2I). In contrast to cultures with live cells (control, Supplementary Figure 2G-2I), 44% of the mouse macrophages showed evidence of engulfing apoptotic MCF-7 cells or apoptotic bodies. The presence of PI-labeled material in the mouse macrophages indicates that apoptotic-cell derived DNA could be rapidly (3h) phagocytized into macrophages in our co-culture model. Previous studies, with mouse and rat phagocytic cells, have demonstrated that apoptotic-cell derived DNA can be integrated into the chromosomes of these cells [28, 45].

### Changes in the migratory and proliferative abilities of mouse peritoneal macrophages after phagocytosis of apoptotic tumor cells

**Figure 2:**
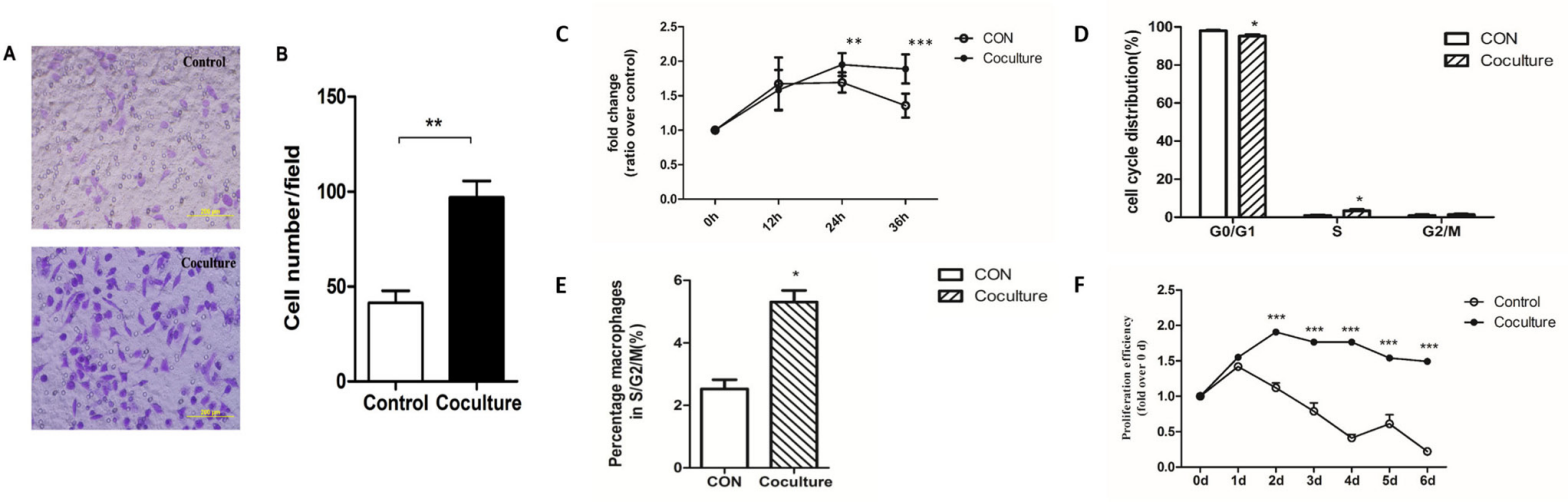
Increased migratory and proliferative abilities in macrophages that have ingested cancer cells A Transwell migration assay was used to assay the migratory ability of macrophages that had or had not phagocytized apoptotic MCF-7 cells. **(A)** Representative microphotographs of migrating cells seen in control (no phagocytosis) or coculture (phagocytosis) cells. **(B)** Quantitative analysis of the numbers of cells on the lower surface of the membrane in the transwell plates. Membranes were fixed and dyed using 0.1% Crystal Violet and number of cells observed in each field was counted. Bars represent the average numbers of cell observed in 5 fields, ** p<0.01. **(C)** Changes in the numbers of macrophages that had (Coculture) or had not (CON) phagocytized apoptotic MCF-7 cells over 36 hours measured by the MTS assay. **(D)** Quantification of the percentages of cells in different phases of the cell cycle determined by flow cytometry. **(E)** Quantification of the percentage of macrophages in S/G2/M phases of the cell cycle by flow cytometry. Results shown are typical of the three independent experiments. **(F)** Change in the numbers of macrophages that had (Coculture) or had not (CON) phagocytized apoptotic MCF-7 cells over 6 days measured by the MTS assay. Data represent means ± S.E. (n=3). * p<0.05, ** p<0.01, *** p<0.001.

To determine whether the phenotype of macrophages change after ingestion of apoptotic tumor cells we examined their migratory and proliferative abilities. We used transwell chambers to examine migration [46, 47]. Macrophages that had or had not phagocytized apoptotic MCF-7 cells were collected, resuspended in serum-free medium, and inoculated into the upper chamber of a transwell chamber. The lower chamber contained macrophage media supplemented with 20% serum. After 24 hours, cells on the lower side of the compartment membrane were fixed, stained with 0.1% crystal violet, and counted under a microscope. A statistically significantly higher number of cells (96.93 ± 8.7 cells per field) were observed for macrophages that phagocytized apoptotic MCF-7 cells compared to macrophages that had not phagocytized apoptotic MCF-7 cells (41.4 ± 6.4 cells per field) (Figure 2A-2B). Our results show that macrophages that have phagocytized apoptotic MCF-7 cells have increased migratory ability compared with control cells.

To examine proliferation, mouse macrophages that had or had not engulfed apoptotic tumor cells were seeded into 96-well plates and their proliferation was measured at 12-hour intervals [48]. After 24h of culture, the number of macrophages per well was significantly higher for those that had engulfed apoptotic MCF-7 cells compared to control cells, and this increase was maintained until 36h (Figure 2C). To further examine the increased proliferative ability, we used flow cytometry analysis to determine whether changes in the distributions of cells within the cell cycle had occurred to macrophages that had engulfed apoptotic tumor cells. As shown in Figure 2D ,a significant decrease in the number of cells in the G0/G1 phase and a significant increase in cells in the S phase was observed for macrophages that had engulfed apoptotic MCF-7 cells. A similar pattern is seen when the number of macrophages in the S/G2/M phases was examined (Figure 2E). As primary macrophages are not immortal, or proliferate indefinitely, we used the MTS method [49, 50] to monitor survival over a one-week period (Figure 2F). Our results show that the number of untreated macrophages begin to decrease after 1 day, with only about 10% of the cells being viable after 6 days (Figure 2F). In contrast, proliferation was observed within 2 days for macrophages that had engulfed apoptotic MCF-7 cells, with little cell death observed even after 6 days (Figure 2F). These results show that both migratory and proliferative ability of macrophages change after engulfing apoptotic MCF-7 cancer cells.

### Human M2 type macrophages incorporate apoptotic cancer cell-derived DNA into their nuclei

Human M2 type macrophages, like mouse M2 macrophages, possess strong phagocytotic and pro-tumor activities [51–53]. To examine whether human macrophages could phagocytize DNA derived from apoptotic cancer cells we differentiated human monocyte derived macrophages (hMDMs) into the M2 phenotype using IL-4 [17]. CD163, which belongs to the scavenger receptor family, was used as a marker to identify M2 macrophages [23–25]. As shown in Supplementary Figure 3A-3D, macrophages stimulated by IL-4 show a macrophage-like appearance and express CD163 after IL-4 stimulation.

**Figure 3:**
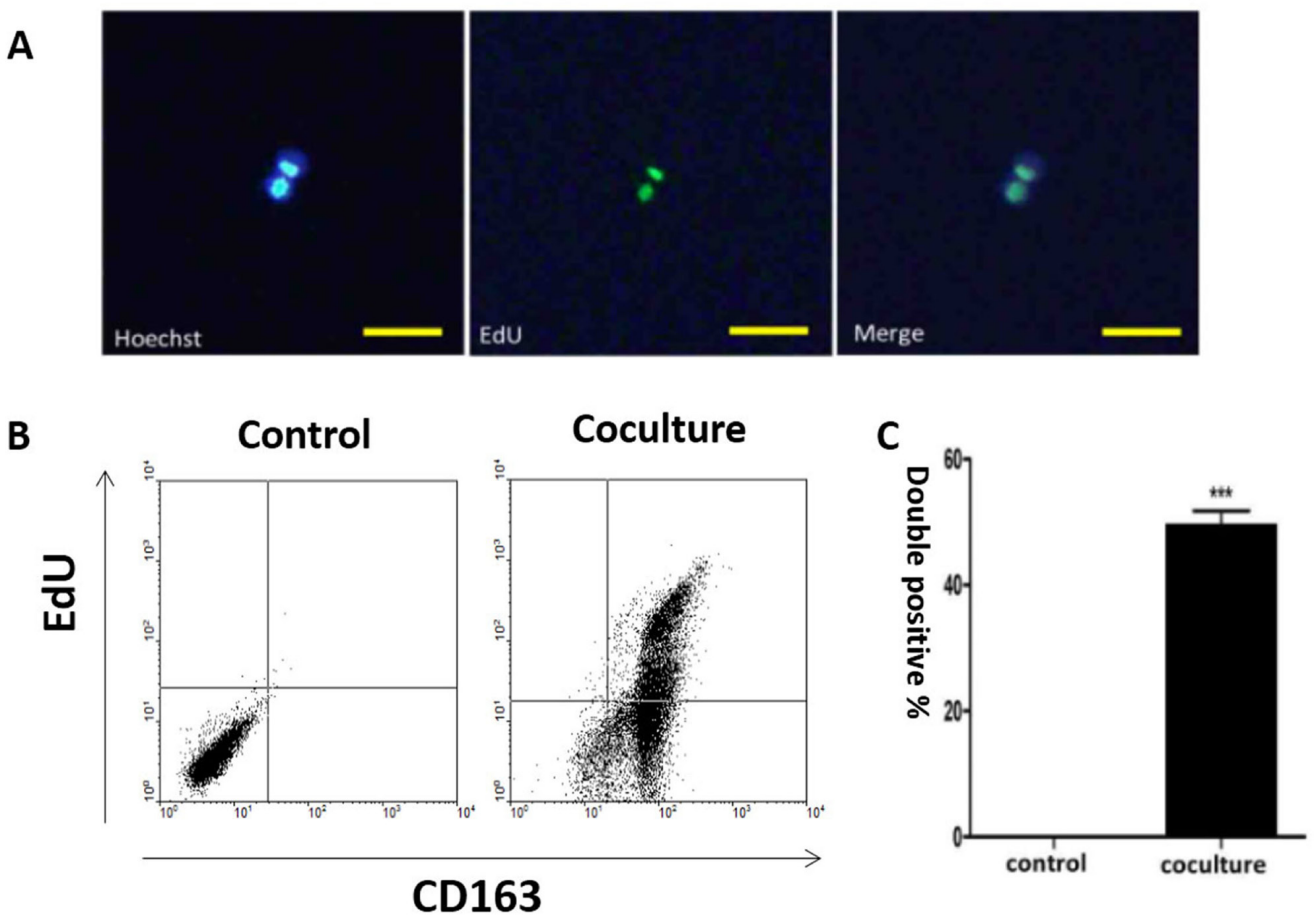
Co-localization of tumor-derived DNA and macrophage nuclei Human macrophages were co-cultured with apoptotic MCF-7 cells. Tumor-derived DNA in MCF-7 cells was labeled with EdU while macrophages were identified by CD163 positivity. **(A)** Immuno-staining of tumor-derived DNA (EdU, green) and hMDM nuclei (Hoechst, blue), with the merged image shown on the right. Scale bar, 100μm. **(B)** Flow cytometry analysis of EdU positive cells for control (macrophages alone)) and cocultured (macrophages and apoptotic MCF-7 cells) cells. Upper right part of the panel is EdU positive hMDMs. **(C)** Quantification of the results shown in (B). Results are typical of three independent experiments. Data represent mean ± S.E. (n=3). ***p< 0.001 vs. Control group.

Apoptotic bodies can be phagocytized by human macrophages [28, 45, 54]. To follow the fate of internalized DNA in our cells, we labeled the genomes of MCF-7 cells with 5-Ethynyl-2’-deoxyuridine (EdU) before inducing apoptosis (Supplementary Figure 4). The presence of MCF-7 derived EdU within macrophages was detected one week after co-culture with the labelled MCF-7 cells, where the label co-localized with the nucleus (Figure 3A). Detection of EdU positive tumor DNA in the nuclei of human macrophages suggests that it was incorporated into the nuclei. EdU positive macrophages were then examined by flow cytometry, which showed that they retained the M2 phenotype as indicated by CD163 and EdU double positivity (Figure 3B-3C).

**Figure 4:**
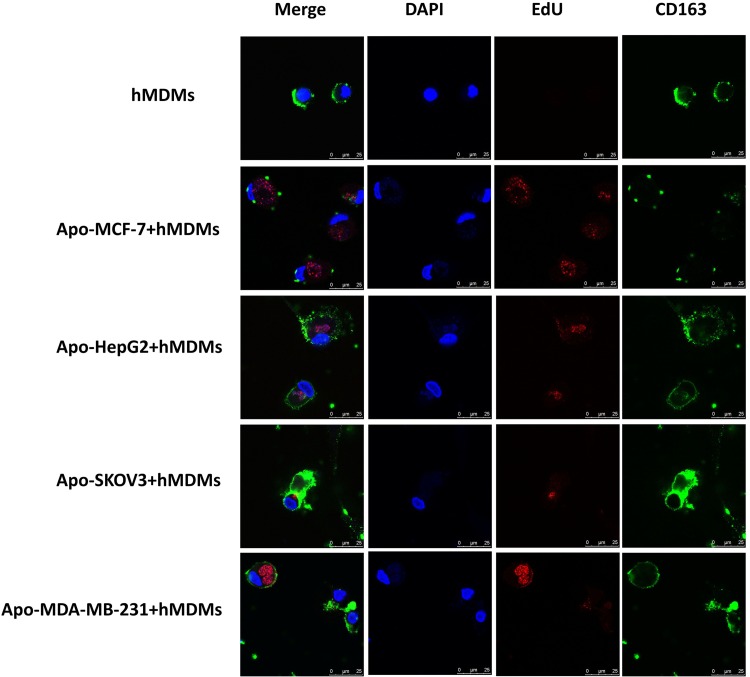

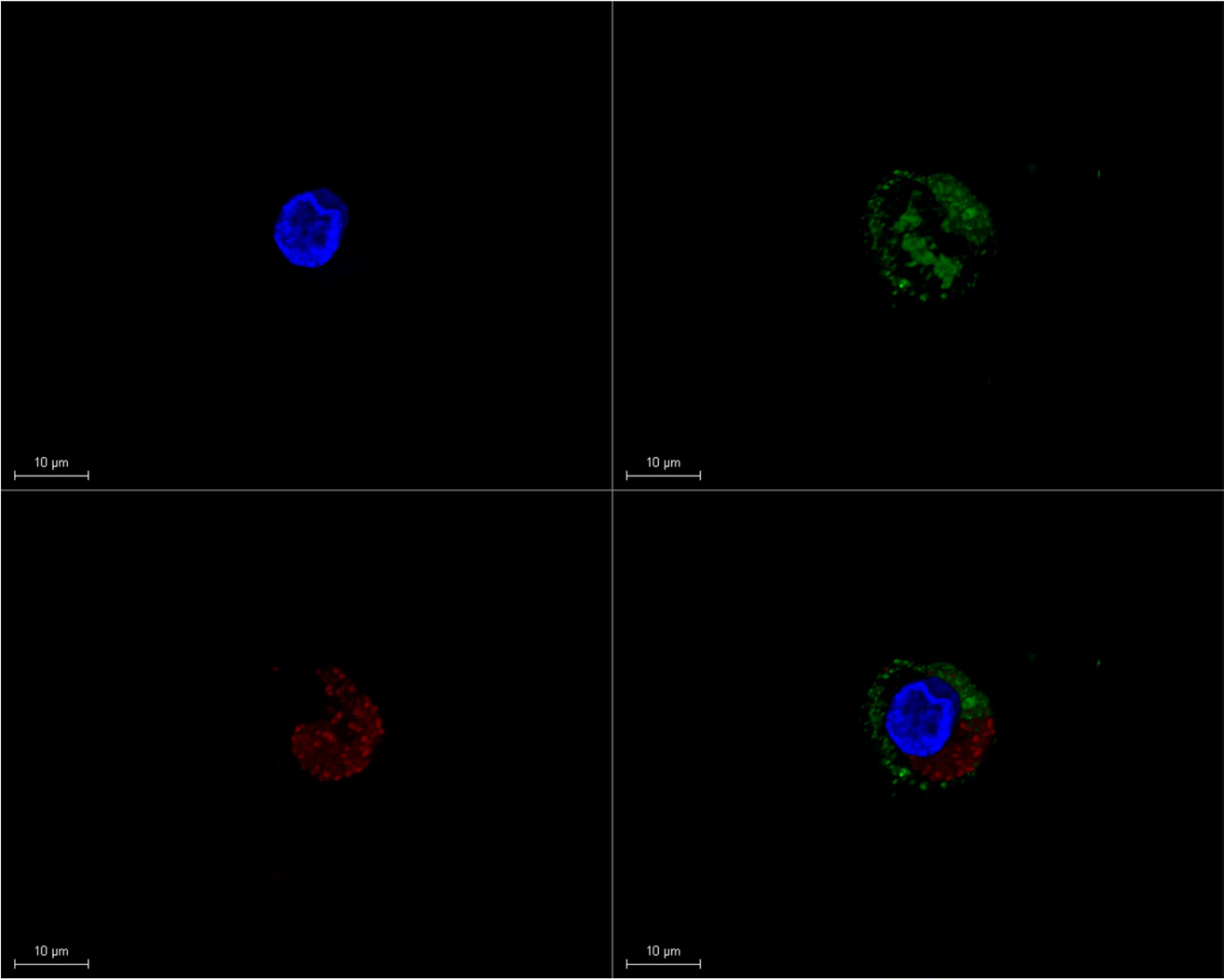
Confocal microscopy analysis and 3D reconstruction of CD163 and EdU expression in hMDMs co-cultured with different apoptotic tumor cells Apoptotic tumor-derived DNA from different types of tumor cells was labeled with EdU (red) prior to induction of apoptosis by H_2_O_2_. hMDMs were identified by CD163 (green) positivity. In this figure, hMDMs were enriched from a healthy subject using Ficoll-Hypaque density gradient centrifugation. **(A)** hMDMs cultured by themselves (as negative control) or with cancer cell line cells that had been induced to have apoptosis (cell lines labeled on left). Cell nuclei were stained with DAPI (blue), apoptotic DAN with EdU (red) and hMDM by CD163 (green) with the merged image on the left. The presence of EDU in the cytoplasm of hMDMs indicated that the hMDMs had engulfed debris from an apoptotic tumor cell. Scale bar, 25μm. **(B)** A single visual field for the co-culture of macrophages and apoptotic MDA-MBA231 cells was selected for 3D reconstruction to give a clearer and more stereoscopic image. Scale bar, 10μm.

To confirm this phenomenon with other types of tumor cells, we labelled the DNA of other cell line cells (SKOV-3, HepG2 and MDA-MB231) with EdU, induced apoptosis and co-cultured with macrophages. After co-culture of apoptotic SKOV-3, HepG2 and MDA-MB231 for three days, EdU staining could be detected in the macrophages, which retain CD163 positivity (Figure 4A and Supplementary Figure 5A). Furthermore, stereoscopic images of visual fields of macrophages that had phagocytized a MDA-MBA231 cell were examined for 3D reconstruction (Figure 4B) with a dynamic map of an image shown in Supplementary Figure 5B.

As shown above, we successfully established an *in vitro* apoptosis-phagocytosis model for both mouse and human macrophages. Our results demonstrate that macrophages bearing the M2 phenotype not only phagocytize dead cells, but can also incorporate tumor-derived DNA into their nuclei. This incorporated DNA might then impact the phenotype of these macrophages, including changing expression of genes and proteins.

### Macrophages transform into epithelial tumor-like cells and gain stem-like properties

**Figure 5:**
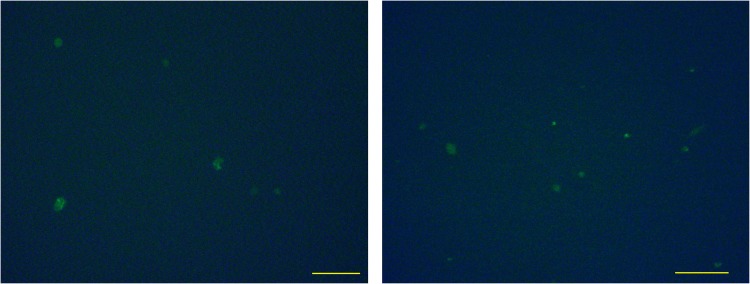
Expression of genes transferred from apoptotic cells to hMDMs MCF-7 cells were transfected with a GFP expressing plasmid and then induced to undergo apoptosis and co-cultured with hMDMs. GFP expression in macrophages after co-culture with apoptotic MCF-7 cells transfected with a GFP expressing plasmid. Two fields of view are shown. Scale bar, 100μm.

Our data suggest that macrophages gained M2-like properties after phagocytizing apoptotic cancer cells. Previous studies, in mice and rats, demonstrated that DNA from apoptotic cells could be integrated into the genome of the phagocytizing cells [28, 45]. As human macrophages appear to incorporate tumor DNA into their nuclei (Figure 3A), we investigated whether these cells utilize the incorporated DNA. For this experiment, we induced apoptosis in MCF-7 cells transfected with a GFP expressing plasmid [45]. After co-culture of these apoptotic cells with macrophages, GFP expression was observed in the macrophages (Figure 5), indicating that GFP was transferred from the dead MCF-7 cells to these cells. Taken together with Figure 3A and a previous study [45], this suggests that apoptotic tumor cells conferred macrophages with novel features through horizontal gene transfer.

**Figure 6:**
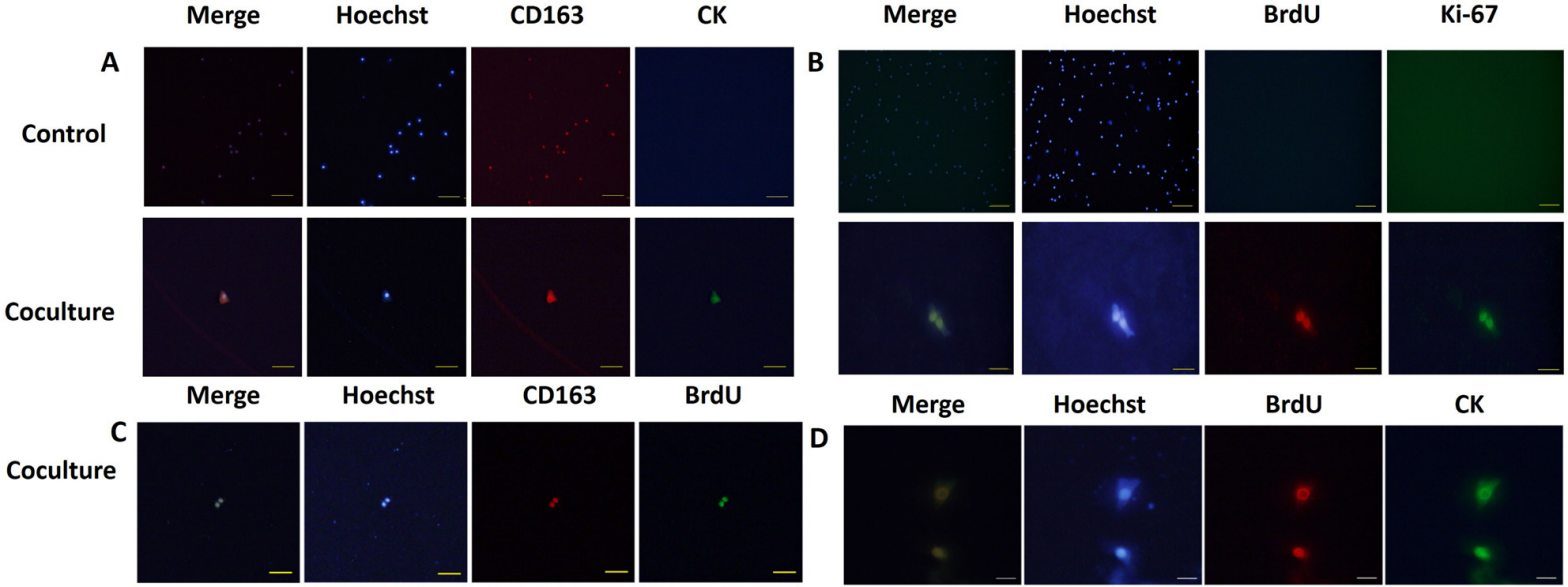
hMDMs reutilize tumor DNA and transform into epithelial-proliferative tumor-like cell retaining M2 marker expression (**A**) hMDMs were co-cultured with apoptotic MCF-7 cells and stained for epithelial markers (CD163, red) and markers of proliferation (CK, green). Nuclei were counterstained stained with Hoechst (blue). Merged images are shown at the left. Control cells were not co-cultured with apoptotic cancer cells. Results show that control hMDMs harbor no CK staining and were incapable of proliferation. The co-culture group shows hMDMs cells expressing both CK and CD163. Fluorescence microscope image of one double-positive cell. Scale bar represents 100μm. **(B)** The control group is hMDMs co-culture with tumor cells stained for Ki-67 (green) and BrdU (red) and nuclei stained with Hoecht (blue). Merged image is shown on the left. Control cells were not co-cultured with apoptotic cancer cells. Results show that the control hMDMs harbor no Ki-67 and BrdU. Scale bar for control cells is 500μm. Co-culture group shows staining for Ki-67 and BrdU. Scale bar for coculture cells is 50μm. **(C)** Staining for CK (green), BrdU (red) and nuclei (Hoechst, blue). Scale bar, 50μm. **(D)** Staining for CD163 (red), BrdU (green) and nuclei (Hoechst, blue). Scale bar, 200μm.

As the MCF-7 breast cancer cell line is of epithelial origin [55] we sought to determine whether the co-cultured macrophages gained epithelial tumor-like cell properties. Pan-cytokeratin (CK) was used as a marker for epithelial identity. By immunofluorescence, co-cultured macrophages were found to be positive for CK expression, while retaining expression of the M2 marker CD163 (Figure 6A). No positive signal for CK was detected in control macrophages (Figure 6A). These results suggest that DNA from the apoptotic cancer cells play a role in new protein expression in macrophages. Since our co-cultured macrophages have acquired epithelial characters from the dead tumor cells, we queried whether they had acquired other functions, such as proliferation, from the cancer cells. To quantify proliferation, we added BrdU to the culture media and examined expression of the proliferation marker Ki67, both of which were examined by immunofluorescence. Strikingly, Ki67 and BrdU double-positive cells were detected in the co-cultured macrophages, although the detection rate was very low (Figure 6B). Occasionally, we observed two adjacent positive cells, suggesting that they were generated by a recent cell division event. The identity of the BrdU expressing macrophages was examined and was found to be CD163 positive (Figure 6C).

Next, we investigated whether the proliferative ability and the epithelial character of the macrophages was gained concomitantly by examining BrdU and CK co-localization. Intriguingly, a few macrophages expressing CK also showed BrdU incorporation (Figure 6D). No positive signals for the proliferation markers were detected in control macrophages (Figure 6B). These results, together with our previous results, allowed us to conclude that not only could macrophages phagocytize dead tumor cells but they could also reuse tumor DNA to gain expression of epithelial markers and initiate the cell cycle to allow proliferation. Macrophages, thus, took advantages of tumor DNA to reshape into progenitor or stem-like cell. Further study is needed to dissect how the tumor derived genetic material acts on the macrophages after phagocytosis, including whether this DNA is integrated into the genome or indirectly derepresses silenced genetic elements.

**Figure 7:**
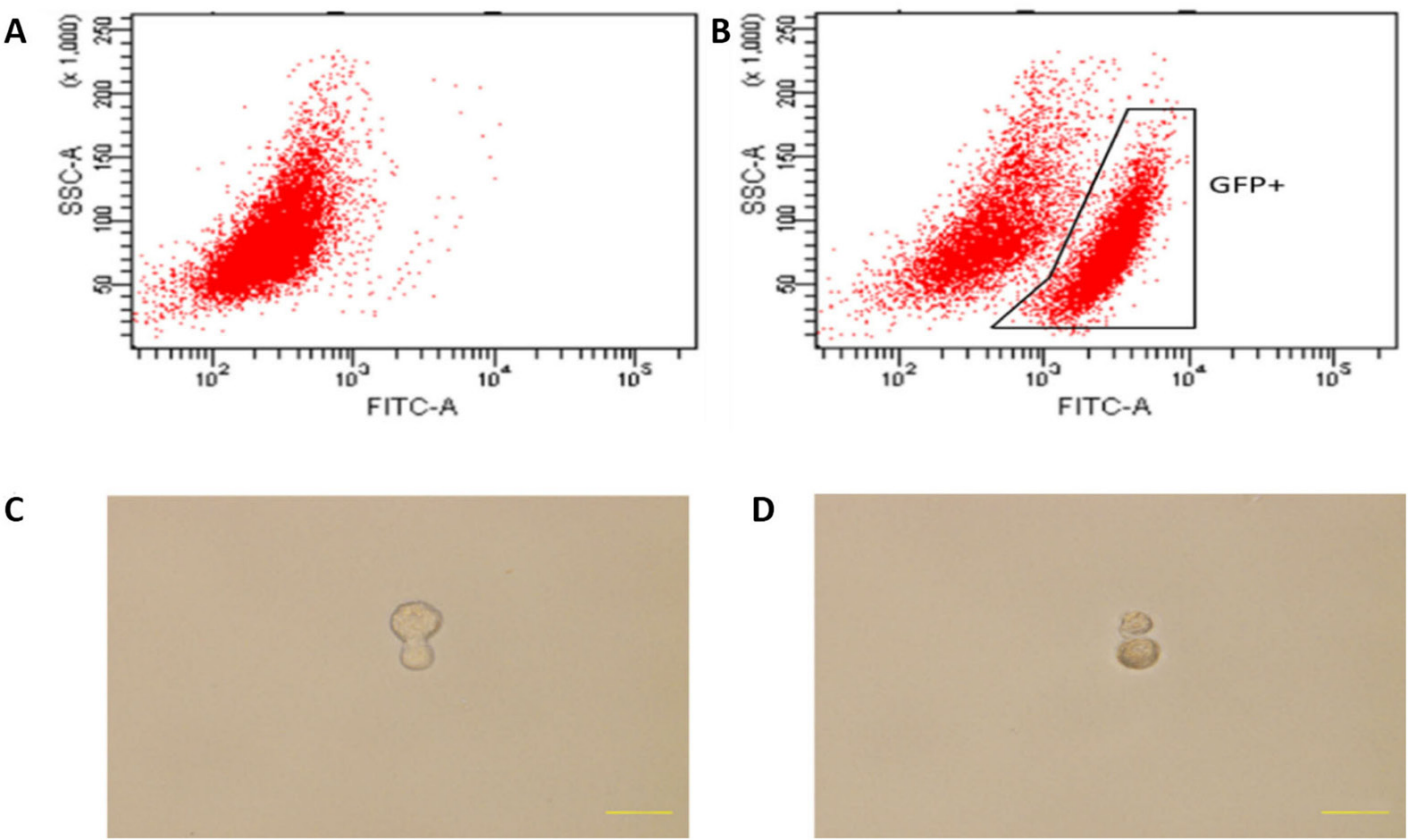
Cell division of co-cultured hMDMs in serum-free media **(A), (B)** Flow cytometry activated sorting of GFP-positive cells. (A) hMDMs cultured alone. (B) hMDMs co-cultured with apoptotic GFP expressing MCF-7 cells. **(C)** Mitotic division of a cell 3 days after cell sorting. **(D)** Mitotic division into two cells after 5 days.

**Figure 8:**
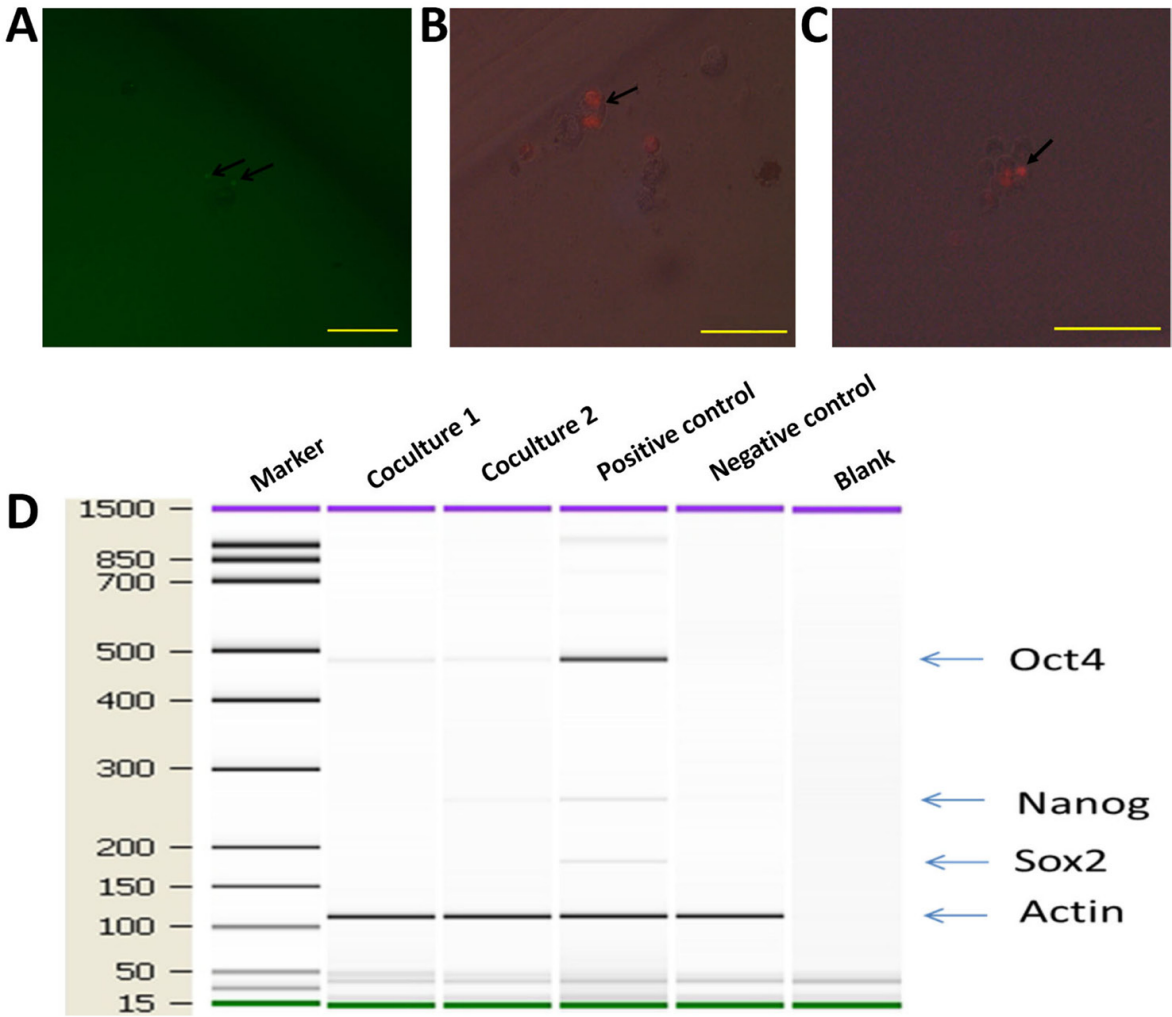
hMDMs not only proliferate but also give rise to mammospheres in serum-free media hMDMs with the Premo Fucci Cell Cycle Sensor that had engulfed apoptotic MCF-7 cells were sorted by flow cytometry and cultured in chemically defined media. **(A)** GFP positive cells inside a cell sphere indicate that these cells were in the G2-S phase. Scale bar, 100μm. **(B)** Cell undergoing mitosis. Two RFP spots inside a cell indicate ongoing cell division. Scale bar, 50μm. **(C)** Cell sphere with mitosis occurring in the core. Scale bar, 100μm. **(D)** Expression ofstem cell marker genes in co-cultured macrophages. Marker shown on the left is the 15-1500bp ladder. Stem cell markers examined were *Oct4*, *Sox2* and *Nanog*. The internal reference gene was β-actin.

Since co-cultured macrophages expressed GFP (Figure 5), FACS (Fluorescence Activated Cell Sorting) was conducted to select GFP expressing cells (Figure 7A-7B), which were then placed into single cells of a 96-well ultra-low attachment plate containing chemically defined serum-free media. Cells were cultured for 3-5 days, and examined for cell division (Figure 7C-7D). To monitor the proliferation status of these cells we employed the Premo Fucci Cell Cycle Sensor, which combines Cdt1 with RFP and geminin with GFP. In the G1 phase of the cell cycle, geminin is broken down and thus only the Cdt1 tagged with RFP can be visualized [56], thus cells in the G1 phase will have red fluorescent nuclei. We sorted 1000 macrophages (co-cultured with apoptotic MCF-7), with 5 sorted cells put in each well of 24-well ultra-low attachment plates, into chemically defined serum-free media containing the cell cycle sensor. By visual inspection, we observed that the number of cells increased over the next few days and that GFP, as well as RFP, positive cells were responsible for this growth in 11 of the 1000 cells (Figure 8A-8B). We also detected the formation of two spheres, with cell proliferating occurring in the core of these spheres after one week of culture in serum-free media (Figure 8C). To further characterize these stem-like cells, we analyzed the expression of a series of stem cell-associated genes in both sphere-derived and control cells. Expression of stem cell-associated genes was found in the sphere-derived but not in the control cells (Figure 8D). Taken together, these results indicate that macrophages bearing the M2 phenotype reutilize DNA, derived from apoptotic tumor cells after phagocytosis of apoptotic tumor cells, to transform into stem-like cells.

### Mononuclear phagocytes isolated from the blood of cancer patients express tumor markers

**Figure 9:**
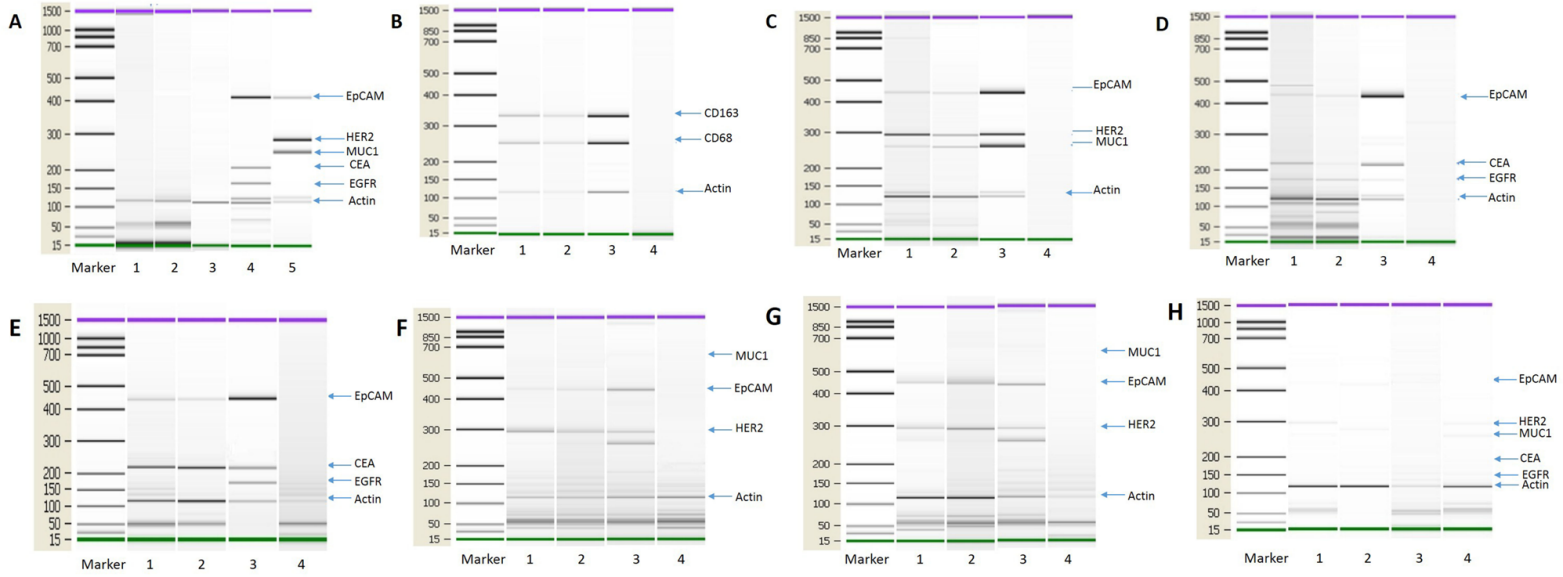
Tumor transcripts in monocytes/macrophages from the blood of cancer patients **(A)** Control test of the immunomagnetic beads. Blood cell samples from healthy subjects were separated using the immunomagnetic beads and analyzed for tumor marker gene expression. No tumor marker genes were detected in cells from healthy humans. Lanes 1: Breast cancer CTC isolation immunomagnetic beads test from healthy humans (n=10). Lane 2: Colon cancer CTC isolation immunomagnetic bead test from healthy humans (n=10). Lane 3: Monocyte/macrophage isolation immunomagnetic bead test from healthy humans (n=10). Lane 4: HCT-8 cell line, as positive control, Lane 5: MCF-7 cell line, as positive control. (**B**) PCR detection of monocyte/macrophage marker genes used for enrichment. Lane 1: CTC immunomagnetic bead depletion followed by monocyte/macrophage immunomagnetic bead enrichment; Lane 2: RosetteSep depletion followed by monocyte/macrophage immunomagnetic bead enrichment; Lane 3: THP-1 cell line, as positive control; Lane 4: Negative control (no template). **(C)** PCR detection of tumor gene transcripts from breast cancer patients. Lane 1: CTC immunomagnetic bead depletion followed by monocyte/macrophage immunomagnetic bead enrichment; Lane 2: RosetteSep depletion followed by monocyte/macrophage immunomagnetic bead enrichment; Lane 3: MCF-7 cell line, as positive control; Lane 4: Negative control (no template). N=12. **(D)** PCR detection of tumor gene transcripts from colon cancer patients. Lane 1: CTC immunomagnetic bead depletion followed by monocyte/macrophage immunomagnetic bead enrichment; Lane 2: RosetteSep depletion followed by monocyte/macrophage immunomagnetic bead enrichment; Lane 3: HCT-8 cell line, as positive control; Lane 4: Negative control (no template). N=8. **(E)** PCR detection of transcripts for tumor cells and monocyte/macrophages from colon cancer patients. Lane 1: CTC immunomagnetic bead enrichment; Lane 2: After CTC immunomagnetic bead enrichment followed by monocyte/macrophage immunomagnetic bead enrichment; Lane 3: HCT-8 cell line, as positive control; Lane 4: Negative control (no template). N=12. **(F)** PCR detection of transcripts for tumor cells and monocyte/macrophages from breast cancer patients. Lane 1: CTC immunomagnetic bead enrichment; Lane 2: After CTC immunomagnetic bead enrichment followed by monocyte/macrophage immunomagnetic bead enrichment; Lane 3: MCF-7 cell line, as positive control; Lane 4: Negative control (no template). N=12. **(G)** PCR detection of transcripts for tumor cells and monocyte/macrophages from ovarian cancer patients. Lane 1: CTC immunomagnetic bead enrichment; Lane 2: After CTC immunomagnetic bead enrichment followed by monocyte/macrophage immunomagnetic bead enrichment; Lane 3: SKOV-3 cell line, as positive control; Lane 4: Negative control (no template). N=12. **(H)** PCR detection of transcripts for tumor cells and monocyte/macrophages from healthy subjects. Lane 1: CTC immunomagnetic bead enrichment for breast cancer markers; Lane 2: CTC immunomagnetic bead enrichment for colon cancer markers; Lane 3: CTC immunomagnetic bead enrichment for ovarian cancer markers; Lane 4: After the above three CTC immunomagnetic bead enrichments followed by monocyte/macrophage immunomagnetic bead enrichment. N=12.

Based on our *in vitro* experimental results, we investigated whether monocyte/macrophages from cancer patients might also contain tumor-compromised cells. To test this speculation, we isolated monocyte/macrophages from cancer patients and interrogated them for the presence of tumor marker gene expression by PCR. Colorectal and breast cancer patients were selected for this analysis in light of their hematogenous metastatic propensity [57, 58]. To enrich for monocyte/macrophage and reduce the presence of CTCs (circulating tumor cells), we employed immunomagnetic beads [2, 3]. CTC depletion beads and monocyte/macrophage enrichment beads were established. The reliability and feasibility of this approach was validated (Figure 9A). To test the identity of the tumor and monocyte/macrophage cells (Figure 9B) we used the marker genes listed in Supplement Table 1. The expression of a number of tumor markers was detected in a subset of the monocyte/macrophages isolated from the blood of cancer patients (Figure 9C-9D), including both breast (n=12) (Figure 9C) and colorectal cancer (n=8) (Figure 9D). We also examined whether these macrophages express epithelial markers. In this experiment, CTCs and macrophages were selected using CTC enrichment and monocyte/macrophage enrichment beads from patients with breast, ovarian and colorectal cancer and the expression of epithelial marker genes were detected by PCR. We first isolated CTC using CTC enrichment beads from the peripheral blood of cancer patients and then macrophages were isolated from the CTC enriched cells using monocyte/macrophage enrichment beads. Macrophages isolated through this process express epithelial markers (Figure 9E-9H). These results, together with those shown in Figure 1, indicate that monocyte/macrophages in the circulation of cancer patients contain tumor-derived material, and thus may define a new type of circulating tumor cell.

## DISCUSSION

Our study provides new insight into the relationship between macrophages and cancer as well as a new theory for the origin of some CSCs and CTCs. *In vivo*, apoptosis of tumor tissue is a natural process and can be initiated in many ways: deregulated cell proliferation leading to lack of nutrients, chemotherapy or radiotherapy causing direct tumoricidal effect, and immune attack [19, 22, 38]. In the early stages of tumor development, macrophages, as a component of the immune system, prime themselves, as the M1 type, to kill tumors and antagonize the suppressive activities of TAMs [19, 20, 22, 38]. M1 macrophages also amplify the TH1 response, providing positive feedback in antitumor activity [19, 22]. The microenvironment of tumor contains multiple types of signals [59, 60] and macrophages can react with cytokines derived from tumors as well as T cells to acquire a polarized M2 phenotype [61, 62].

Preliminary research has suggested that high numbers of TAMs is associated with a poor prognosis [30, 40, 53]; however, TAMs have yet to be precisely phenotyped or categorized [63]. TAMs have diverse phenotypes, which partly stems from differences in their tumor type source, donors and isolation techniques [64, 65]. Some clinical studies have found that CD163, a marker shared with M2 macrophages, correlate with disease outcome with higher levels of CD163 associated with a worse patient outcome [66, 67]. We selected CD163 as a marker to identify macrophages.

A possible explanation for mononuclear phagocytes gaining pluripotency and tumor-like phenotype is through cell fusion [11, 31–33, 68, 69]. However, other explanations are possible. Mononuclear phagocytes, as part of the innate immune system, defend against invasion by microorganisms such as viruses by engulfing them [70]. Once cells or viruses have been engulfed by macrophages there is a potential that horizontal gene transfer (HGT) could occur with engulfed DNA being incorporated into the macrophage genome. Transfer of HIV DNA from apoptotic bodies to macrophage genomes has been observed [54, 71]. Similarly, DNA from Epstein Bar virus and *H. pylori* (*Helicobacter pylori*) has been detected in nasopharynx and gastric cancers, respectively [72, 73]. The frequent interaction between microorganism and monocyte/macrophages may increase the susceptibility to HGT by mononuclear phagocytes.

Mononuclear phagocytes are versatile and plastic cells that respond to environmental influences through the expression of distinct transcriptional programs and functions [19, 22, 38]. High plasticity helps these cells adapt to complex micro-environmental conditions such as cancer [74–76]. The M1 and M2 macrophages are extremes of a continuum, and there is a host of intermediate states that result from the exposure to micro-environmental signals [77]. During the production of these different states, the genomes of macrophages constantly switch on and off the expression of multiple genes. Our study revealed that interactions between macrophages and tumor cells leads to the emergence of a pro-tumor phenotype by macrophages that probably resulted from the integration of tumor DNA into their genomes. Under natural condition, we postulate there might be time points where the genome of macrophages is weakly protected, potentially due to its plastic or viral-primed state, where tumor DNA can be integrated into its genome [78].

A macrophage-to-tumor transition provides a better interpretation for five existing cancer developmental phenomena. First, it explains why the liver, bone, lung and lymph glands are likely targets for metastasis. These organs typically release large amounts of SDF-1 (stromal cell derived factor-1), a chemoattractant that attracts immune cell expressing CXCR4 on their surface, such as monocytes/macrophages [79, 80]. Second, as a member of the immune system, macrophages are identified as “one of us” by other immune cells. Once they apostatize, they remain unchanged in their outer appearance, while having a tumor genome. This double identity makes it easier for a tumacrophage to escape immunosurveillance [81, 82]. Third, the epithelial-to-mesenchymal transition theory explains how an epithelial tumor cell can acquire motility to metastasize and invade. Tumacrophages can easily move due to their inherent nature as macrophages and have mesenchymal features. As we detected epithelial marker in the transformed macrophage, EMT could be rewritten as mesenchyme-being-epithelized [83]. Fourth, research has demonstrated that a possible reason why tumor treatments often have poor outcomes is that macrophages phagocytize dead tumor cell, and thus become transformed and repopulate the tumor sites *in situ* and metastasize to distant organs. Fifth, TAMs harbor the ability to establish extracellular matrix [22, 38]. As soon as macrophages are transformed, they not only possess matrix building ability, but also begin to do so.

**Figure 10:**
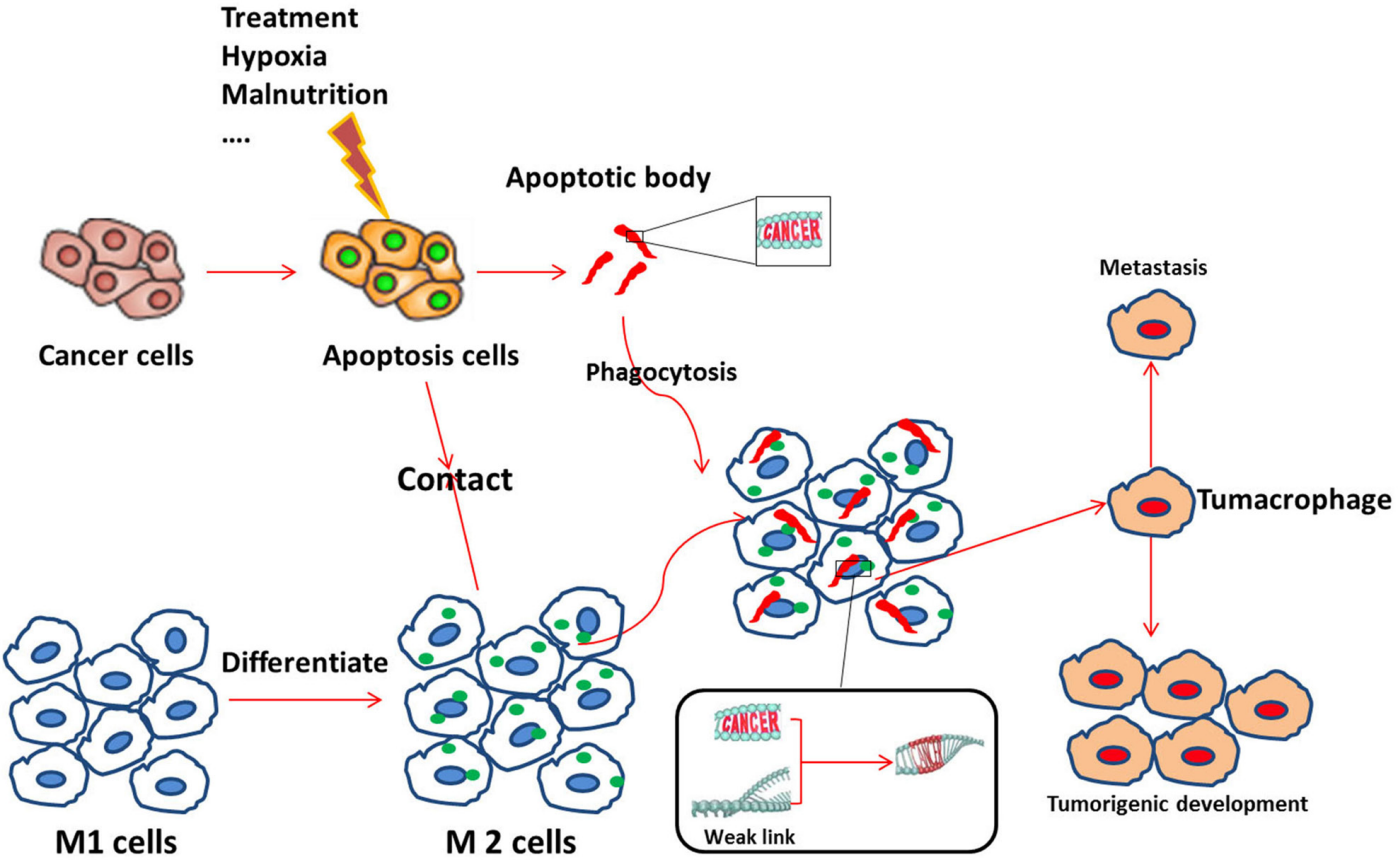
Possible mechanism for the emergence of tumacrophages First, an external (e.g., treatment, hypoxia) or internal (e.g., tumor malnutrition owing to outgrowth of the nutritient supply) stress induces apoptosis of tumor cells. Apoptotic signals, such as apoptotic bodies, attract M1 macrophage and stimulate them to differentiate into the M2 type. M2 macrophages then scavenge cellular debris, including apoptotic bodies, which may contain fragments of the cancerous cell genomes. Dynamic differentiation of macrophages responding to the complicated tumor microenvironment may yield weak links (open chromatin) in the cellular genomes due to the constant switching on and off of genes. Foreign genetic elements from the cancer gene-containing apoptotic bodies are then incorporated or trans-activate some silenced gene(s). Accumulation of changes at critical moments transforms macrophages into tumacrophages that bear both a macrophage phenotype and a cancerous core. This unique double identity of the tumacrophages allows these cells to travel around the body, escaping immunesurveillance, and give rise to metastasis after finding favorable residence in tissues such as the lung, liver, or bone.

In summary, we demonstrate that macrophages phagocytize apoptotic tumor cells, with tumor DNA being incorporated into the nuclei of the macrophages. These tumor-DNA carrying macrophages appear to become compromised and transition into tumor stem cells, with tumorigenic ability, but retain cell surface macrophage markers. Based on the aforementioned findings, we propose the hypothesis presented in Figure 10: After macrophages phygocytize dead cancer cells, genetic material derived from the dead cancer cells confer new cellular abilities or identity on the macrophages through trans-activation or direct integration. The new cell, which we name tumacrophge, harbor tumorigenic abilities, including cancer stem cell properties, but retains its immune privilege inherited from the macrophage. The unique identity and ability is the bane of the cancer-related phenomenon, yielding immune evasion, metastasis, and tolerance to chemo- and radio-therapy. This might explain why the clinical practice of chemo- or radio-therapy results in cancer metastasis that are more aggressive. Cancer cells can travel around inside the body by getting a free ride in macrophages. In the future, we will investigate the mechanism of this transition, including locating specific DNA segments that trigger these changes.

## MATERIALS AND METHODS

### Isolation of PBMCs (peripheral blood mononuclear cells) from tumor patients and healthy subjects

The Institutional Review Board at Peking University Health Science Center approved all human and animal experiments with authorization number IRB00001052-16022. All experiments were carried out in accordance with approved guidelines. Informed consent was obtained from all human subjects. For isolation of PBMCs, 3ml venous blood was collected from cancer patients, including breast, cervical, ovarian, endometrial and pancreatic cancer, and healthy human volunteers. The mononuclear fraction was isolated after density gradient centrifugation (400g for 20 min) using Ficoll Paque-plus (GE healthcare). Subsequently, balanced salt solution (BSS) was added to wash the mononuclear cells, and centrifuged at 100g for 10 min. The supernatant was removed and the cell pellet was resuspended in RPMI-1640 media (Gibco), without Fetal Bovine Serum (FBS) (Biological Industry). Monocytes were separated from other white blood cells by allowing them to attach to the dishes for 2 hours. Monocytes were plated at a density of 2.5 ×10^6^/ml in glass confocal dishes (D35-20-0-N, Cellvis).

### Confocal microscopy analysis

Prior to immunofluorescence staining, monocytes were allowed to adhere to the glass bottom of confocal dishes for 2 hours. Cells were then washed 3 times (5 min each) with PBS (Phosphate Buffer Saline), fixed in 4% paraformaldehyde at room temperature for 15 min and then washed again in PBS 3 times (5 min each). Cells were blocked in 5% bovine serum albumin in PBS-T (PBS +0.2% Triton X-100) for 1 hour at room temperature. Primary antibodies (CD163, ab87099, Abcam and EpCAM, ab20160, Abcam) were diluted to 1:40 in blocking solution. 200uL of the primary antibody solutions were added to each dish, and cells were incubated overnight at 4°C. After incubation, cells were washed 3 times (5 min each) with PBS. All subsequent steps were performed in the dark. 200μl of secondary antibodies (TRITC goat anti-mouse IgG, ZF-0313, FITC goat anti-rabbit IgG, ZF-0313, 1:50 dilution in blocking solution) was added to each dish, and incubated for 60 min at room temperature. After incubation, excess secondary antibodies were removed and 200μl of DAPI (C0065, Solarbio) was added to each dish and incubated for 10 min at room temperature in the dark. Cells were then washed 3 times (5 min each) with PBS and observed under a confocal microscope (Leica).

### Isolation of primary mouse macrophages and hepatocytes

Female ICR mice, 6-8 weeks old, were acclimatized in the Experimental Animal Center of Peking University Health Science Center for one week. Mice were sacrificed by cervical dislocation and macrophages were collected by peritoneal lavage with PBS followed by lavage fluid collection through a Pasteur pipette. Cells were washed three times with PBS and cultured in Dulbecco’s Modified Eagle Medium (DMEM) (Gibco) supplemented with 10% (v/v) Fetal Bovine Serum (FBS) (Biological Industry) and 1% antibiotic solution (100 units/ml penicillin, 100μg/ml streptomycin) in a humidified atmosphere of 5% CO_2_ at 37°C for 2h. Non-adherent cells were discarded by washing with PBS after the initial culture, and adherent cells were collected and considered to be peritoneal macrophages for the following studies. Mouse primary hepatocytes were generated by the two-step *in situ* collagenase perfusion method [84]. Isolated hepatocytes were cultured in collagen-treated 6-well plates containing DMEM with 10% FBS as described above.

### Induction and detection of apoptosis

MCF-7, MDA-MB231, SKOV3 and HepG2 cell lines were from our lab and were cultured with DMEM containing 10% FBS in a humidified atmosphere of 5% CO_2_ at 37°C. Cell number was adjusted to 5×10^6^ or 10×10^6^ before induction of apoptosis Cells were cultured for 24h with hydrogen peroxide (H_2_O_2_) at concentrations of 0mM, 0.1mM, 0.2mM and 0.3mM. 10×10^6^ cells were only tested at the 0.3mM concentration. After incubation with H_2_O_2_, cells were washed 3 times with PBS, trypsinized with 0.25% trypsin, collected and then labeled with Annexin V for 30min at 4°C in the dark, followed by labeling with 5ul propidium iodide (PI) for 5min at room temperature in the dark. Rates of apoptosis were deduced by flow cytometry using a BD FACSCalibur.

### Apoptosis-phagocytosis study

Complete apoptosis of MCF-7 cells was induced using H_2_O_2_. Any material from the original culture was removed by washing three times with PBS and dead cells were collected for labelling with PI, with a final concentration of 50ug/ml, for 5 min in room temperature, in the dark. PI-labeled cells were added into a co-culture with mouse peritoneal macrophages for 3h at MCF-7: macrophage ratios of 1:1 and 2:1. Dead MCF-7 cells were washed away with PBS, and the macrophages were digested with lidocaine-EDTA. Cells were then incubated with CD11b-FITC (BD Pharmingen) for 30 min at 4°C. Samples were analyzed with a BD FACSCalibur and confirmed by fluorescence microscope (Olympus).

For human macrophages, 5-ethynyl-2´-deoxyuridine (EdU) was added into the medium, at a final concentration of 5uM, for labeling the MCF-7 cells during the logarithmic growth phase. EdU labeling was for 48h. Labeling efficiency was measured using Click-iT EdU Alexa Fluor 594 Imaging Kit (Life Technologies). Apoptosis in the EdU labeled MCF-7 cells was induced as described above, and the apoptotic cells were added to co-cultures with hMDMs at a ratio of 5:1. Culture media was changed after 48 hours, to remove dead MCF-7 cells, and then changed every 3 days. Following one week of co-culture, the phagocytosis efficiency of EdU was analyzed using CD163-PE and Click-iT Plus EdU Alexa Fluor 647 Flow Cytometry Assay Kit (Life Technologies). The location of EdU was detected using a Cell-Light EdU Apollo 488 (or 567) *In Vitro* Imaging Kit (Ribo Bio)

### Cell tracing with CellTracker

MCF-7 and primary mouse hepatocytes were washed 2 times with PBS and then digested with 0.25% trypsin. Digested cells were collected by centrifugation at 1000rpm for 5min. Mouse peritoneal macrophages were also washed twice with PBS. CellTracker Probes (Life Technologies) was diluted with DMEM with 10% FBS to generate working solutions. Green CMFDA was used to label macrophages and red CMTMR for labeling MCF-7 or primary hepatocytes. Labeling was conducted in a humidified atmosphere of 5% CO_2_ at 37°C for 30min. After labeling, cells were washed 3 times and the media was changed to CellTracker free DMEM complete media. MCF-7 and hepatocyte cells were added into macrophage-culture plates at a ratio of 2:1 (MCF-7/hepatocyte: macrophage). Cells were co-cultured for 3h and then fixed with 4% paraformaldehyde for 15min. Fixed cells were washed 3 times with PBS and observed under a fluorescence microscope (Olympus). Results were recorded by photography and interpreted using ImageJ (National Institutes of Health).

### Measurement of migratory ability using transwell plates

Macrophages were collected, resuspended in serum-free medium, and inoculated into the upper chamber of a transwell plate. The lower chambers of the plates were supplemented with 20% serum-containing macrophage culture medium. After incubation for 24 hours, cells on the lower part of the compartment membrane were fixed and stained with 0.1% crystal violet, counted under a microscope.

### Cell proliferation and analysis of cell cycle phase

The proliferative rates of mouse peritoneal macrophages that had or had not engulfed apoptotic tumor cells were quantified with the MTS assay using CellTiter^®^ 96 Aqueous One Solution Cell Proliferation Assay (Promega Biotech Co., Ltd., Madison, WI, USA) according to the manufacturer’s instructions. Briefly, cells were seeded at the density of 10,000 cells/well into 96-well plates. At the time of assay, 20μL MTS was added to each well and incubated for 4h in a humidified, 5% CO_2_ atmosphere. Proliferation was measured at 12-hour intervals and quantified by measuring absorbance at 490nm using a Vmax microplate reader (Bio-Rad, Hercules, CA, USA). Cell cycle phase was analyzed by flow cytometry using a BD FACSCalibur.

### Isolation of human monocyte-derived macrophages and flow cytometry analysis

For the isolation of human monocyte-derived macrophages (hMDMs), venous blood was collected from healthy human volunteers and the mononuclear fraction isolated after density gradient centrifugation (400g for 20 min) using Ficoll Paque-plus (GE healthcare). Cells were plated at a density of 2.5×10^6^/ml in RPMI-1640 (Gibco) without FBS for 2 hours. Non-adherent cells were discarded after 2h and replaced with RPMI-1640 supplemented with 10% (v/v) FBS incubated for 7d, with media changed every 3d. To obtain M2 polarized cells, 40 ng/ml IL-4 (Peprotech) was added after the initial culture for 72h [17]. Macrophages were then detached from the plates using 2.0 mg/ml Dispase II (Sigma) [15]. For differentiation analysis, cells were incubated with CD14-FITC (BD Pharmingen) and CD163-PE (BD Pharmingen). Cell samples were then run on a BD FACSCalibur (BD, USA).

### Immunofluorescent staining

To detect epithelial markers, macrophagres were fixed using 4% paraformaldehyde, washed twice with PBS and permeabilized with 0.5% Triton X100 in PBS. Cells were then washed 3 times with PBS and incubated with the blocking solution (10% goat serum in PBS) and then incubated with antibodies against Cytokeratin (CK) (1:20, mouse anti-human; Abcam) and CD163 (1:40, rabbit anti-human; Abcam) for 16h, washed 3 times with PBS for 15 minutes, and finally incubated with secondary antibodies (Alexa Fluor 488, 1:200, goat anti-mouse; and Alexa Fluor 594, 1:200, goat anti-rabbit; Jackson Immuno Research) and Hoechst 33342 for 1h. Samples were washed extensively with PBS and imaged by fluorescence and confocal microscopy.

To detect proliferation, 5-Bromo-2-deoxyUridine (BrdU) was added to the macrophage culture media to 5uM and cells were cultured for 48h. Cells were then fixed using 4% paraformaldehyde, washed twice with PBS and permeabilized with 0.5% Triton X100 in PBS. Cells were then washed 3 times with PBS and incubated with blocking solution (10% goat serum in PBS) and then incubated with antibodies against Ki-67 (1:50, mouse anti-human; Abcam), CD163 (1:40, rabbit anti-human; Abcam), BrdU (1:100, rat anti-human; Abcam), Cytokeratin (1:20, mouse anti-human; Abcam) for 16h, washed 3 times with PBS 15 minutes, and finally incubated with secondary antibodies (Alexa Fluor 488, 1:200, goat anti-mouse; Alexa Fluor 594, 1:200, goat anti-rabbit; TRITC, 1:100, goat anti-rat; FITC, 1:100, goat anti-rat; Jackson Immuno Research) and Hoechst 33342 for 1h. Samples were washed extensively with PBS and detected by fluorescence microscopy.

### Functional labeling of MCF-7 cells by GFP transfection

The GFP expression vector pCAG-GFP vector was purchased from Addgene. MCF-7 cells were trypsinized and resuspended in HEPES buffered saline (HBS) with 6mM glucose at 4 ×10^6^/ml. Cells were incubated at 4°C for 10 min with 10ug of vector. After incubation, cells were electroporated using a Gene Pulser Xcell (Bio-rad) at room temperature, with conditions set as 960μF, 230v. Cells were then cultured for 12h before induction of apoptosis.

### Detection of stemness in co-cultured macrophages

GFP-expressing macrophages were sorted using BD FACSAriaIII (BD) and placed into 96-well ultra-low attachment plate (Corning) containing chemically defined serum-free media [85, 86] consisting of SensiCell 1640 media (Life Technologies) supplemented with human recombinant EGF (20ng/ml; Life Technologies), bFGF (20ng/ml; Life Technologies), HGF (20ng/ml; Life Technologies), B27 (Life Technologies), human recombinant insulin (5μg/ml; MP), heparin (4μg/ml; Sigma), BSA (0.4%; Sigma), Hydrocortisone (0.48μg/ml; Stemcell), and antibiotic-antimycotic (Life Technologies).

MCF-7 cells were induced into apoptosis and co-cultured with macrophages. After co-culture, a limiting dilution assay was performed on the macrophages to yield 5 cells per well in 24-well ultra-low attachment plates (Corning) containing the growth media described above. The Premo FUCCI Cell Cycle Sensor was added into the plates in a 1ul volume according to the manufacture’s instruction. For the mammosphere formation group, cells were processed with the RNeasy Plus Micro Kit (QIAGEN) for RNA isolation. mRNA was converted into cDNA using the Sensiscript RT Kit (QIAGEN). PCR primers for stem-cell-specific markers include *Oct4*, *Sox2*, and *Nanog*, and the PCR reactions were performed using the Multiplex PCR Kit (QIAGEN). PCR products were analyzed with an Agilent Bioanalyzer 2100.

### Sorting of monocyte/macrophages in cancer patients

Circulating tumor cells were isolated from patients diagnosed with colon, breast and ovarian cancer using magnetic bead kits from Beijing N&N Genetech Company. For breast cancer, these kits used antibodies against EpCAM (Abcam), EGFR (Abcam) and HER2 (Abcam), for colon cancer the antibodies were against EpCAM (Abcam), EGFR (Abcam) and CK20 (Abcam), while for ovarian cancer the antibodies were against EpCAM (Abcam), MUC1 (Abcam) and HER2 (Abcam). Monocyte/macrophages were isolated using similar magnetic beads (from Beijing N&N Genetech) with antibodies against CD14 (Abcam), CD163 (Abcam) and CD68 (Abcam) [26, 27]. Dynabeads M-450 Tosylactivated (Life Technologies) were labeled with the antibodies according to the manufacturer’s protocol.

To deplete CTCs for targeting monocyte/macrophage, we first incubated the blood samples with CTC isolation beads. After the beads were separated from the blood, we added monocyte/macrophage isolation magnetic beads. The RosetteSep CD45 depletion Kit (Stemcell) was used to separate CTC from monocyte/macrophages. Samples were processed according to the manufacture’s protocol and CTCs were depleted. The remaining cell pellet was lysed with red blood cell lysis buffer (Leagene) to release monocyte/macrophage and then we used isolation beads for positive isolation to enrich for monocyte/macrophages. Isolated cells were employed for mRNA isolation using the Dynabeads mRNA Direct Kit (Life Technologies). mRNA was converted into cDNA as described above with the Sensiscript RT Kit. For PCR analysis, primer sets for the breast and ovarian cancer gene markers EpCAM, MUC-1 and HER2 were designed. A similar primer sets of marker genes for colon cancer included EpCAM, CEA and EGFR [2, 3], while primers for CD163 and CD68 were used to detect monocyte/macrophages. β-actin was used as an endogenous reference gene for each group.

## SUPPLEMENTARY MATERIALS FIGURES, TABLE AND VIDEO





## References

[1] da Silva SD, Hier M, Mlynarek A, Kowalski LP, Alaoui-Jamali MA (2012). Recurrent oral cancer: current and emerging therapeutic approaches. Front Pharmacol.

[2] Yu M, Stott S, Toner M, Maheswaran S, Haber DA (2011). Circulating tumor cells: approaches to isolation and characterization. J Cell Biol.

[3] Alix-Panabieres C, Pantel K (2013). Circulating tumor cells: liquid biopsy of cancer. Clin Chem.

[4] Kim MY, Oskarsson T, Acharyya S, Nguyen DX, Zhang XH, Norton L, Massague J (2009). Tumor self-seeding by circulating cancer cells. Cell.

[5] Pantel K, Brakenhoff RH (2004). Dissecting the metastatic cascade. Nat Rev Cancer.

[6] Wang JC, Dick JE (2005). Cancer stem cells: lessons from leukemia. Trends Cell Biol.

[7] Lapidot T, Sirard C, Vormoor J, Murdoch B, Hoang T, Caceres-Cortes J, Minden M, Paterson B, Caligiuri MA, Dick JE (1994). A cell initiating human acute myeloid leukaemia after transplantation into SCID mice. Nature.

[8] Bonnet D, Dick JE (1997). Human acute myeloid leukemia is organized as a hierarchy that originates from a primitive hematopoietic cell. Nat Med.

[9] Klimp AH, de Vries EG, Scherphof GL, Daemen T (2002). A potential role of macrophage activation in the treatment of cancer. Crit Rev Oncol Hematol.

[10] Vassilopoulos G, Wang PR, Russell DW (2003). Transplanted bone marrow regenerates liver by cell fusion. Nature.

[11] Willenbring H, Bailey AS, Foster M, Akkari Y, Dorrell C, Olson S, Finegold M, Fleming WH, Grompe M (2004). Myelomonocytic cells are sufficient for therapeutic cell fusion in liver. Nat Med.

[12] Fessler E, Borovski T, Medema JP (2015). Endothelial cells induce cancer stem cell features in differentiated glioblastoma cells via bFGF. Mol Cancer.

[13] Blagosklonny MV (2006). Target for cancer therapy: proliferating cells or stem cells. Leukemia.

[14] Munz M, Baeuerle PA, Gires O (2009). The emerging role of EpCAM in cancer and stem cell signaling. Cancer Res.

[15] Nardin A, Abastado JP (2008). Macrophages and cancer. Front Biosci.

[16] Teng MW, Swann JB, Koebel CM, Schreiber RD, Smyth MJ (2008). Immune-mediated dormancy: an equilibrium with cancer. J Leukoc Biol.

[17] Haase J, Weyer U, Immig K, Kloting N, Bluher M, Eilers J, Bechmann I, Gericke M (2014). Local proliferation of macrophages in adipose tissue during obesity-induced inflammation. Diabetologia.

[18] Forssell J, Oberg A, Henriksson ML, Stenling R, Jung A, Palmqvist R (2007). High macrophage infiltration along the tumor front correlates with improved survival in colon cancer. Clin Cancer Res.

[19] Mills CD, Kincaid K, Alt JM, Heilman MJ, Hill AM (2000). M-1/M-2 macrophages and the Th1/Th2 paradigm. J Immunol.

[20] Mantovani A, Bottazzi B, Colotta F, Sozzani S, Ruco L (1992). The origin and function of tumor-associated macrophages. Immunol Today.

[21] Balkwill F, Mantovani A (2001). Inflammation and cancer: back to Virchow?. Lancet.

[22] Sica A, Bronte V (2007). Altered macrophage differentiation and immune dysfunction in tumor development. J Clin Invest.

[23] Harris JA, Jain S, Ren Q, Zarineh A, Liu C, Ibrahim S (2012). CD163 versus CD68 in tumor associated macrophages of classical Hodgkin lymphoma. Diagn Pathol.

[24] Heusinkveld M, van der Burg SH (2011). Identification and manipulation of tumor associated macrophages in human cancers. J Transl Med.

[25] Lau SK, Chu PG, Weiss LM (2004). CD163: a specific marker of macrophages in paraffin-embedded tissue samples. Am J Clin Pathol.

[26] Martin-Fuentes P, Civeira F, Recalde D, Garcia-Otin AL, Jarauta E, Marzo I, Cenarro A (2007). Individual variation of scavenger receptor expression in human macrophages with oxidized low-density lipoprotein is associated with a differential inflammatory response. J Immunol.

[27] Taylor PR, Martinez-Pomares L, Stacey M, Lin HH, Brown GD, Gordon S (2005). Macrophage receptors and immune recognition. Annu Rev Immunol.

[28] Holmgren L, Szeles A, Rajnavolgyi E, Folkman J, Klein G, Ernberg I, Falk KI (1999). Horizontal transfer of DNA by the uptake of apoptotic bodies. Blood.

[29] Hao NB, Lu MH, Fan YH, Cao YL, Zhang ZR, Yang SM (2012). Macrophages in tumor microenvironments and the progression of tumors. Clin Dev Immunol.

[30] Medrek C, Ponten F, Jirstrom K, Leandersson K (2012). The presence of tumor associated macrophages in tumor stroma as a prognostic marker for breast cancer patients. BMC Cancer.

[31] Kerbel RS, Lagarde AE, Dennis JW, Donaghue TP (1983). Spontaneous fusion *in vivo* between normal host and tumor cells: possible contribution to tumor progression and metastasis studied with a lectin-resistant mutant tumor. Mol Cell Biol.

[32] Chakraborty AK, Sodi S, Rachkovsky M, Kolesnikova N, Platt JT, Bolognia JL, Pawelek JM (2000). A spontaneous murine melanoma lung metastasis comprised of host x tumor hybrids. Cancer Res.

[33] Rachkovsky M, Sodi S, Chakraborty A, Avissar Y, Bolognia J, McNiff JM, Platt J, Bermudes D, Pawelek J (1998). Melanoma x macrophage hybrids with enhanced metastatic potential. Clin Exp Metastasis.

[34] Osta WA, Chen Y, Mikhitarian K, Mitas M, Salem M, Hannun YA, Cole DJ, Gillanders WE (2004). EpCAM is overexpressed in breast cancer and is a potential target for breast cancer gene therapy. Cancer Res.

[35] Gastl G, Spizzo G, Obrist P, Dunser M, Mikuz G (2000). Ep-CAM overexpression in breast cancer as a predictor of survival. Lancet.

[36] Konigsberg R, Obermayr E, Bises G, Pfeiler G, Gneist M, Wrba F, de Santis M, Zeillinger R, Hudec M, Dittrich C (2011). Detection of EpCAM positive and negative circulating tumor cells in metastatic breast cancer patients. Acta Oncol.

[37] Bellone S, Siegel ER, Cocco E, Cargnelutti M, Silasi DA, Azodi M, Schwartz PE, Rutherford TJ, Pecorelli S, Santin AD (2009). Overexpression of epithelial cell adhesion molecule in primary, metastatic, and recurrent/chemotherapy-resistant epithelial ovarian cancer: implications for epithelial cell adhesion molecule-specific immunotherapy. Int J Gynecol Cancer.

[38] Mosser DM, Edwards JP (2008). Exploring the full spectrum of macrophage activation. Nat Rev Immunol.

[39] Mantovani A, Polentarutti N, Luini W, Peri G, Spreafico F (1979). Role of host defense merchanisms in the antitumor activity of adriamycin and daunomycin in mice. J Natl Cancer Inst.

[40] Moding EJ, Kastan MB, Kirsch DG (2013). Strategies for optimizing the response of cancer and normal tissues to radiation. Nat Rev Drug Discov.

[41] Ravichandran KS (2010). Find-me and eat-me signals in apoptotic cell clearance: progress and conundrums. J Exp Med.

[42] Elliott MR, Chekeni FB, Trampont PC, Lazarowski ER, Kadl A, Walk SF, Park D, Woodson RI, Ostankovich M, Sharma P, Lysiak JJ, Harden TK, Leitinger N, Ravichandran KS (2009). Nucleotides released by apoptotic cells act as a find-me signal to promote phagocytic clearance. Nature.

[43] Fadok VA, Voelker DR, Campbell PA, Cohen JJ, Bratton DL, Henson PM (1992). Exposure of phosphatidylserine on the surface of apoptotic lymphocytes triggers specific recognition and removal by macrophages. J Immunol.

[44] Li J, Huang CY, Zheng RL, Cui KR, Li JF (2000). Hydrogen peroxide induces apoptosis in human hepatoma cells and alters cell redox status. Cell Biol Int.

[45] Bergsmedh A, Szeles A, Henriksson M, Bratt A, Folkman MJ, Spetz AL, Holmgren L (2001). Horizontal transfer of oncogenes by uptake of apoptotic bodies. Proc Natl Acad Sci U S A.

[46] Wang HQ, Huang LX, Qu MJ, Yan ZQ, Liu B, Shen BR, Jiang ZL (2006). Shear stress protects against endothelial regulation of vascular smooth muscle cell migration in a coculture system. Endothelium.

[47] Wolf K, Friedl P (2006). Molecular mechanisms of cancer cell invasion and plasticity. Br J Dermatol.

[48] McGowan EM, Alling N, Jackson EA, Yagoub D, Haass NK, Allen JD, Martinello-Wilks R (2011). Evaluation of cell cycle arrest in estrogen responsive MCF-7 breast cancer cells: pitfalls of the MTS assay. PLoS One.

[49] Soman G, Yang X, Jiang H, Giardina S, Vyas V, Mitra G, Yovandich J, Creekmore SP, Waldmann TA, Quinones O, Alvord WG (2009). MTS dye based colorimetric CTLL-2 cell proliferation assay for product release and stability monitoring of interleukin-15: assay qualification, standardization and statistical analysis. J Immunol Methods.

[50] O'Toole SA, Sheppard BL, McGuinness EP, Gleeson NC, Yoneda M, Bonnar J (2003). The MTS assay as an indicator of chemosensitivity/resistance in malignant gynaecological tumours. Cancer Detect Prev.

[51] Mantovani A, Biswas SK, Galdiero MR, Sica A, Locati M (2013). Macrophage plasticity and polarization in tissue repair and remodelling. J Pathol.

[52] Mitchem JB, Brennan DJ, Knolhoff BL, Belt BA, Zhu Y, Sanford DE, Belaygorod L, Carpenter D, Collins L, Piwnica-Worms D, Hewitt S, Udupi GM, Gallagher WM (2013). Targeting tumor-infiltrating macrophages decreases tumor-initiating cells, relieves immunosuppression, and improves chemotherapeutic responses. Cancer Res.

[53] Noy R, Pollard JW (2014). Tumor-associated macrophages: from mechanisms to therapy. Immunity.

[54] Spetz AL, Patterson BK, Lore K, Andersson J, Holmgren L (1999). Functional gene transfer of HIV DNA by an HIV receptor-independent mechanism. J Immunol.

[55] Nie S, McDermott SP, Deol Y, Tan Z, Wicha MS, Lubman DM (2015). A quantitative proteomics analysis of MCF7 breast cancer stem and progenitor cell populations. Proteomics.

[56] Sakaue-Sawano A, Kurokawa H, Morimura T, Hanyu A, Hama H, Osawa H, Kashiwagi S, Fukami K, Miyata T, Miyoshi H, Imamura T, Ogawa M, Masai H, Miyawaki A (2008). Visualizing spatiotemporal dynamics of multicellular cell-cycle progression. Cell.

[57] Geng Y, Chandrasekaran S, Agastin S, Li J, King MR (2014). Dynamic switch between two adhesion phenotypes in colorectal cancer cells. Cell Mol Bioeng.

[58] Harvey JR, Mellor P, Eldaly H, Lennard TW, Kirby JA, Ali S (2007). Inhibition of CXCR4-mediated breast cancer metastasis: a potential role for heparinoids?. Clin Cancer Res.

[59] Tang X (2013). Tumor-associated macrophages as potential diagnostic and prognostic biomarkers in breast cancer. Cancer Lett.

[60] Pollard JW (2008). Macrophages define the invasive microenvironment in breast cancer. J Leukoc Biol.

[61] Weigert A, Tzieply N, von Knethen A, Johann AM, Schmidt H, Geisslinger G, Brune B (2007). Tumor cell apoptosis polarizes macrophages role of sphingosine-1-phosphate. Mol Biol Cell.

[62] Wu A, Wei J, Kong LY, Wang Y, Priebe W, Qiao W, Sawaya R, Heimberger AB (2010). Glioma cancer stem cells induce immunosuppressive macrophages/microglia. Neuro Oncol.

[63] Wada N, Zaki MA, Hori Y, Hashimoto K, Tsukaguchi M, Tatsumi Y, Ishikawa J, Tominaga N, Sakoda H, Take H, Tsudo M, Kuwayama M, Morii E, Aozasa K (2012). Tumour-associated macrophages in diffuse large B-cell lymphoma: a study of the Osaka Lymphoma Study Group. Histopathology.

[64] Balkwill F, Charles KA, Mantovani A (2005). Smoldering and polarized inflammation in the initiation and promotion of malignant disease. Cancer Cell.

[65] Pollard JW (2004). Tumour-educated macrophages promote tumour progression and metastasis. Nat Rev Cancer.

[66] Han Q, Shi H, Liu F (2016). CD163(+) M2-type tumor-associated macrophage support the suppression of tumor-infiltrating T cells in osteosarcoma. Int Immunopharmacol.

[67] Park JY, Sung JY, Lee J, Park YK, Kim YW, Kim GY, Won KY, Lim SJ (2016). Polarized CD163+ tumor-associated macrophages are associated with increased angiogenesis and CXCL12 expression in gastric cancer. Clin Res Hepatol Gastroenterol.

[68] Powell AE, Anderson EC, Davies PS, Silk AD, Pelz C, Impey S, Wong MH (2011). Fusion between intestinal epithelial cells and macrophages in a cancer context results in nuclear reprogramming. Cancer Res.

[69] Aractingi S, Kanitakis J, Euvrard S, Le Danff C, Peguillet I, Khosrotehrani K, Lantz O, Carosella ED (2005). Skin carcinoma arising from donor cells in a kidney transplant recipient. Cancer Res.

[70] Cousins FL, Kirkwood PM, Saunders PT, Gibson DA (2016). Evidence for a dynamic role for mononuclear phagocytes during endometrial repair and remodelling. Sci Rep.

[71] Gaiffe E, Pretet JL, Launay S, Jacquin E, Saunier M, Hetzel G, Oudet P, Mougin C (2012). Apoptotic HPV positive cancer cells exhibit transforming properties. PLoS One.

[72] Cao JX, Cui YX, Long ZJ, Dai ZM, Lin JY, Liang Y, Zheng FM, Zeng YX, Liu Q (2010). Pluripotency-associated genes in human nasopharyngeal carcinoma CNE-2 cells are reactivated by a unique epigenetic sub-microenvironment. BMC Cancer.

[73] Kim KJ, Wen XY, Yang HK, Kim WH, Kang GH (2015). Prognostic implication of M2 macrophages are determined by the proportional balance of tumor associated macrophages and tumor infiltrating lymphocytes in microsatellite-unstable gastric carcinoma. PLoS One.

[74] Weitzenfeld P, Meshel T, Ben-Baruch A (2016). Microenvironmental networks promote tumor heterogeneity and enrich for metastatic cancer stem-like cells in Luminal-A breast tumor cells. Oncotarget.

[75] Meacham CE, Morrison SJ (2013). Tumour heterogeneity and cancer cell plasticity. Nature.

[76] Friedmann-Morvinski D (2014). Glioblastoma heterogeneity and cancer cell plasticity. Crit Rev Oncog.

[77] Rhee I (2016). Diverse macrophages polarization in tumor microenvironment. Arch Pharm Res.

[78] Huang J (2013). Horizontal gene transfer in eukaryotes: the weak-link model. Bioessays.

[79] Yamada S, Shimada M, Utsunomiya T, Imura S, Morine Y, Ikemoto T, Mori H, Hanaoka J, Saito Y, Asanoma M, Miyake H (2012). Action of SDF-1/CXCR4 axis in liver metastasis of colorectal cancer. J Clin Oncol.

[80] Amara S, Chaar I, Khiari M, Ounissi D, Weslati M, Boughriba R, Hmida AB, Bouraoui S (2015). Stromal cell derived factor-1 and CXCR4 expression in colorectal cancer promote liver metastasis. Cancer Biomark.

[81] Domschke C, Schneeweiss A, Stefanovic S, Wallwiener M, Heil J, Rom J, Sohn C, Beckhove P, Schuetz F (2016). Cellular immune responses and immune escape mechanisms in breast cancer: determinants of immunotherapy. Breast Care (Basel).

[82] Zhou X, Mao Y, Zhu J, Meng F, Chen Q, Tao L, Li R, Fu F, Liu C, Hu Y, Wang W, Zhang H, Hua D (2016). TGF-β1 promotes colorectal cancer immune escape by elevating B7-H3 and B7-H4 via the miR-155/miR-143 axis. Oncotarget.

[83] Yu M, Bardia A, Wittner BS, Stott SL, Smas ME, Ting DT, Isakoff SJ, Ciciliano JC, Wells MN, Shah AM, Concannon KF, Donaldson MC, Sequist LV (2013). Circulating breast tumor cells exhibit dynamic changes in epithelial and mesenchymal composition. Science.

[84] Seglen PO (1976). Preparation of isolated rat liver cells. Methods Cell Biol.

[85] Yu M, Bardia A, Aceto N, Bersani F, Madden MW, Donaldson MC, Desai R, Zhu H, Comaills V, Zheng Z, Wittner BS, Stojanov P, Brachtel E (2014). Cancer therapy. *Ex vivo* culture of circulating breast tumor cells for individualized testing of drug susceptibility. Science.

[86] Yuan X, Curtin J, Xiong Y, Liu G, Waschsmann-Hogiu S, Farkas DL, Black KL, Yu JS (2004). Isolation of cancer stem cells from adult glioblastoma multiforme. Oncogene.

