# Supplementary material for: Tumacrophage: macrophages transformed into tumor stem-like cells by virulent genetic material from tumor cells
Source: Oncotarget. 2017 Jul 18;8(47):82326–43. doi: 10.18632/oncotarget.19320 (PMC5669893; doi:10.18632/oncotarget.19320)
Supplement: Supplementary file 1 [file oncotarget-08-82326-s001.pdf]

# Tumacrophage: macrophages transformed into tumor stem-like cells by virulent genetic material from tumor cells

## SUPPLEMENTARY MATERIALS

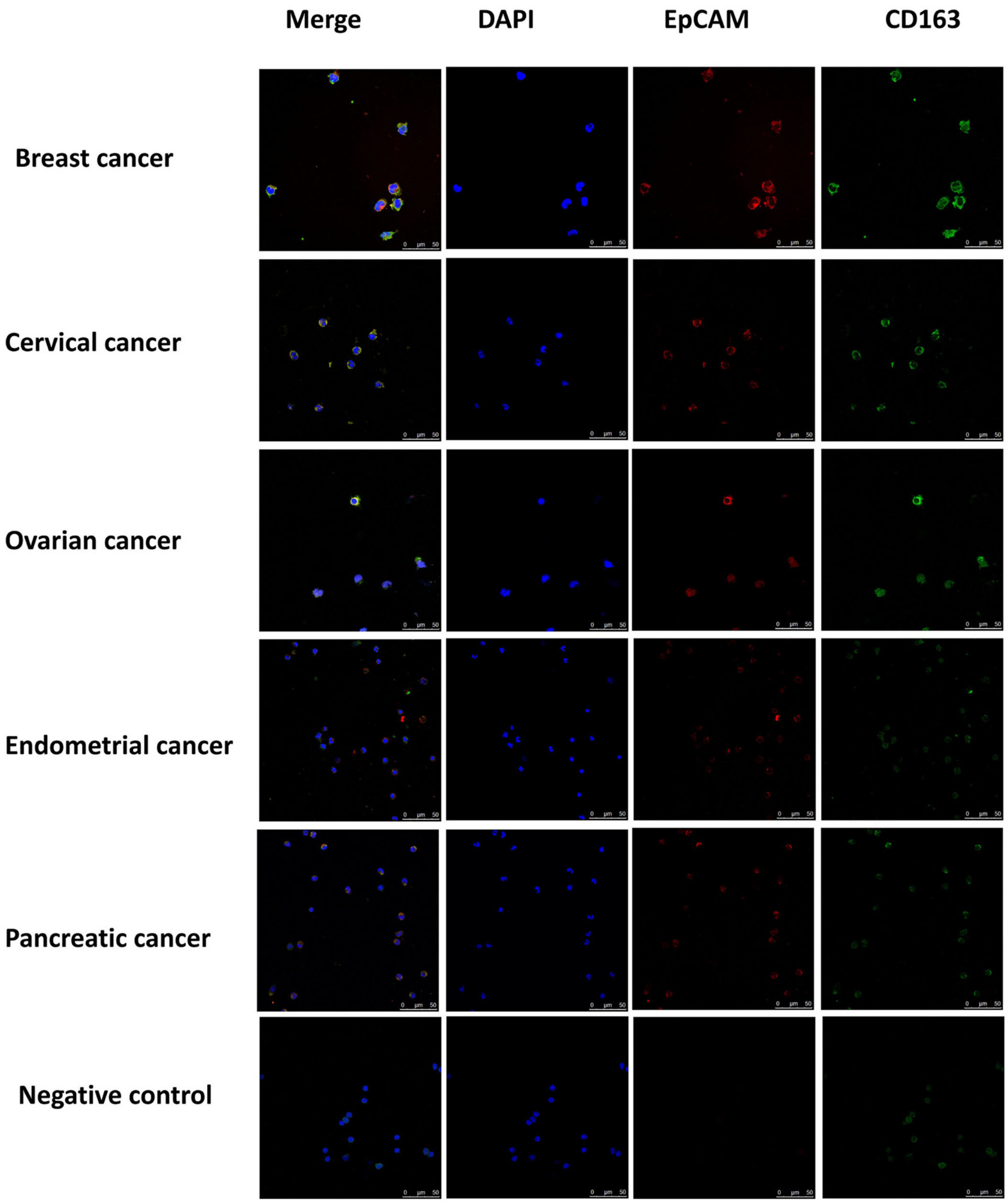


**Supplementary Figure 1: CD163 and EpCAM are co-expressed in monocytes from patients with different types of epithelial tumors.** Immuno-staining of monocytes from patients with breast, cervical, ovarian, endometrial, and pancreatic cancer and a healthy subject, with CD163 labeled in green, EpCAM labeled in red, with nuclei stained with DAPI (blue). A merged figure is shown on the left. Co-localization of CD163 and EpCAM is observed in cells from patients with cancer. Multiple monocytes were observed in each field of view of the microscope, indicating that these cells were not rare. Monocytes from healthy subjects lack EpCAM expression. Scale bar, 50µm.

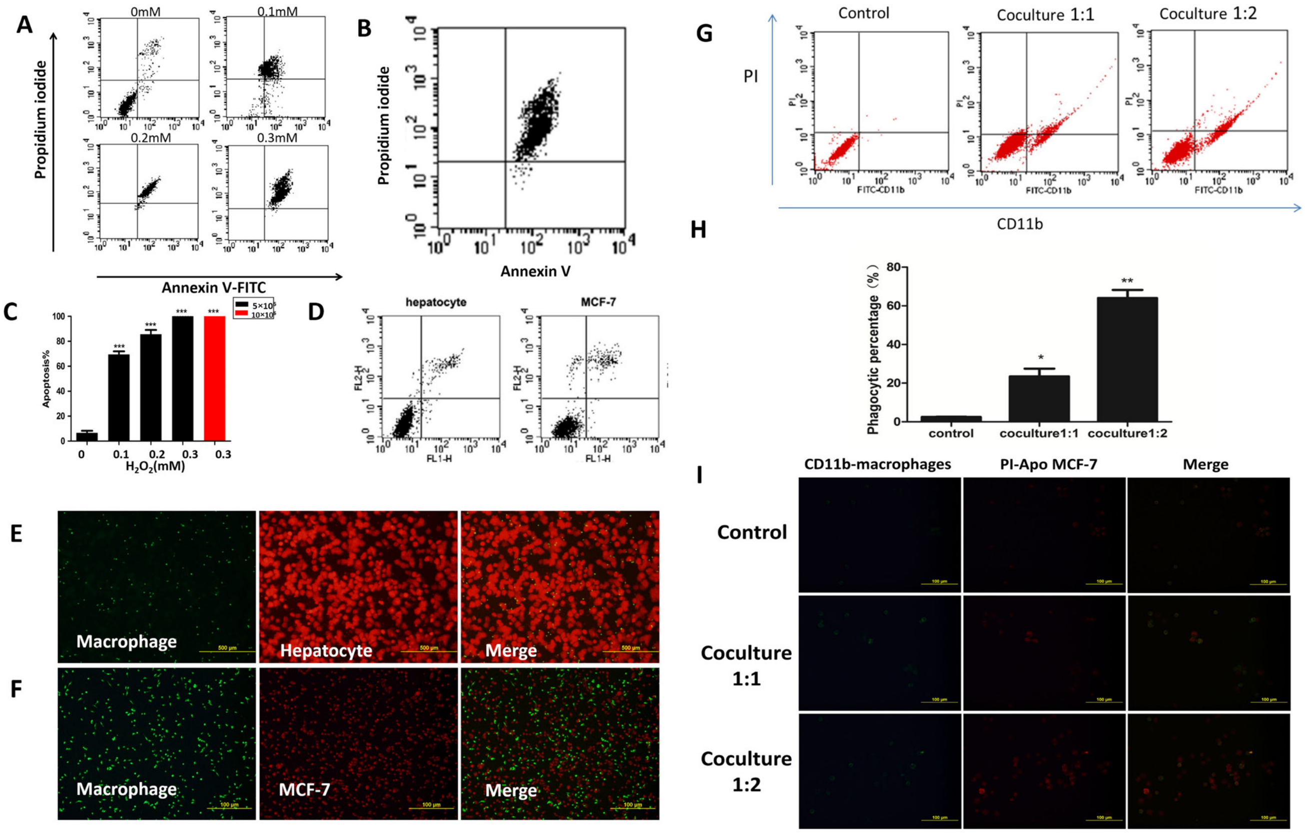


**Supplementary Figure 2: Induction of apoptosis by $H_2O_2$ in MCF-7 cells, and phagocytosis of apoptotic cells by macrophages in co-culture.** MCF-7 cells were treated with different concentrations of $H_2O_2$ for 24h to induce apoptosis. **(A)** Flow cytometry analysis for Annexin V/Propidium Iodide (PI) staining of $5x10^6$ cells at 0, 0.1, 0.2, and 0.3mM $H_2O_2$. **(B)** Flow cytometry analysis of Annexin V/Propidium Iodide (PI) staining of $10x10^6$ cells with 0.3mM $H_2O_2$. **(C)** Quantification of the results from (A) and (B). **(D)** Analysis of the rate of apoptosis in cultured hepatocytes and MCF-7 cells not treated with $H_2O_2$. **(E, F)** Co-culture of CellTracker labeledmacrophages (green) with untreated (E) primary hepatocytes (red) or (F) apoptotic MCF-7 cells (red) for 3h. Scale bar, 100µm. **(G, H, I)** Uptake of apoptotic cell-derived DNA by macrophages. Apoptotic cell-derived DNA was labeled with PI (red) and macrophages were labeled with CD11b (FITC). Macrophages and apoptotic MCF-7 cells were co-cultured at ratios of 1:1 and 1:2 (Macrophage: MCF-7). Controls were non-apoptotic (live) MCF-7 cells. (G) Flow cytometric analysis of co-cultured cell populations for PI (DNA) and CD11b (FITC-CD11b, macrophages). Upper right part of each panel represents macrophages that have phagocytized DNA. (H) Quantitative analysis of (G). Results are typical of three independent experiments. Data represent mean ± S.E. (n=3). $^*p<0.05$, $^{**}p< 0.01$ vs. Control group. (I) Fluorescent microscopic images of co-cultured cells with macrophages immuno-stained for CD11b (green) and DNA from apoptotic MCF-7 cells labeled with PI (red). Scale bar, 100µm.

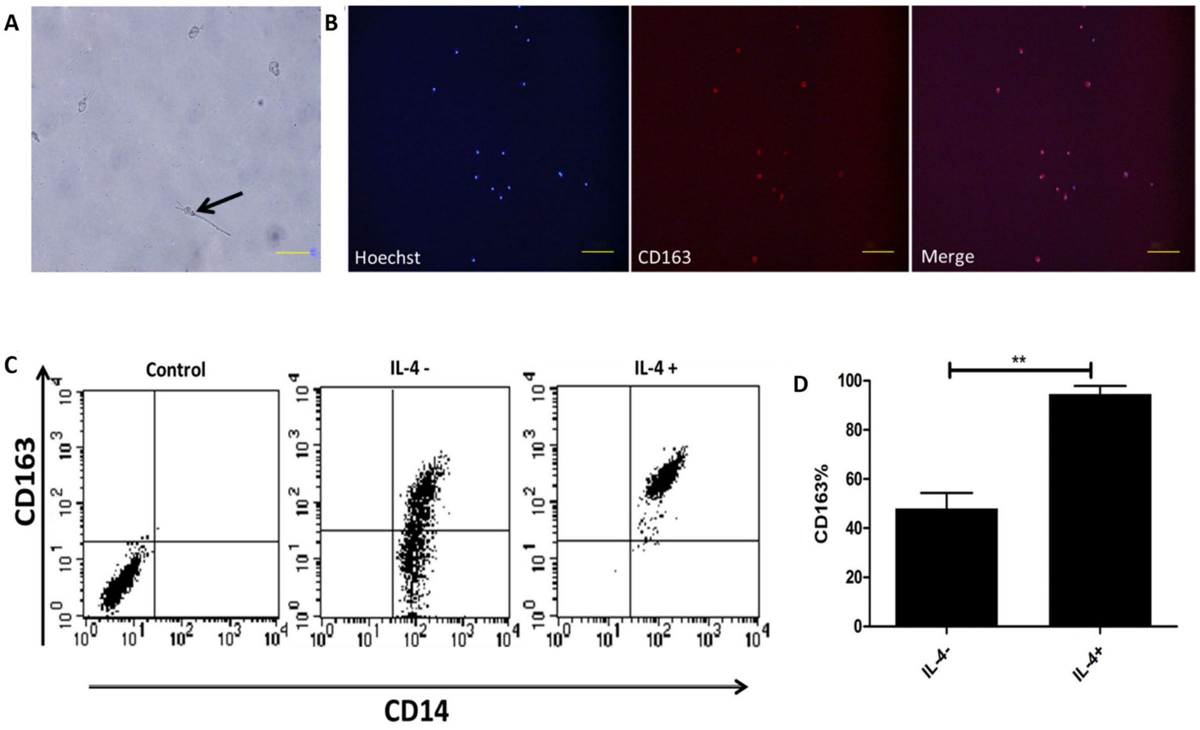


**Supplementary Figure 3: Macrophage-like appearance of hMDMs differentiated with IL-4.** hMDMs express CD163, a marker for the M2 phenotype, after induction by IL-4. **(A)** Representative phase contrast images of monocyte-derived macrophages isolated from blood and after induction by IL-4. Black arrow indicates a representative cell with pseudopod. Scale bar, 200µm. **(B)** Double immunofluorescence staining for CD163 (red) and nuclei (blue) in IL-4 treated human hMDMs. Scale bar, 500µm. **(C)** Flow cytometry analysis of CD163 positive cells. Upper right part of the graph is CD163 expressing hMDMs. **(D)** Quantitative analysis of (C). Results are typical of three independent experiments. Data represent means ± S.E. (n=3). **$p < 0.01$ vs. IL-4- group.

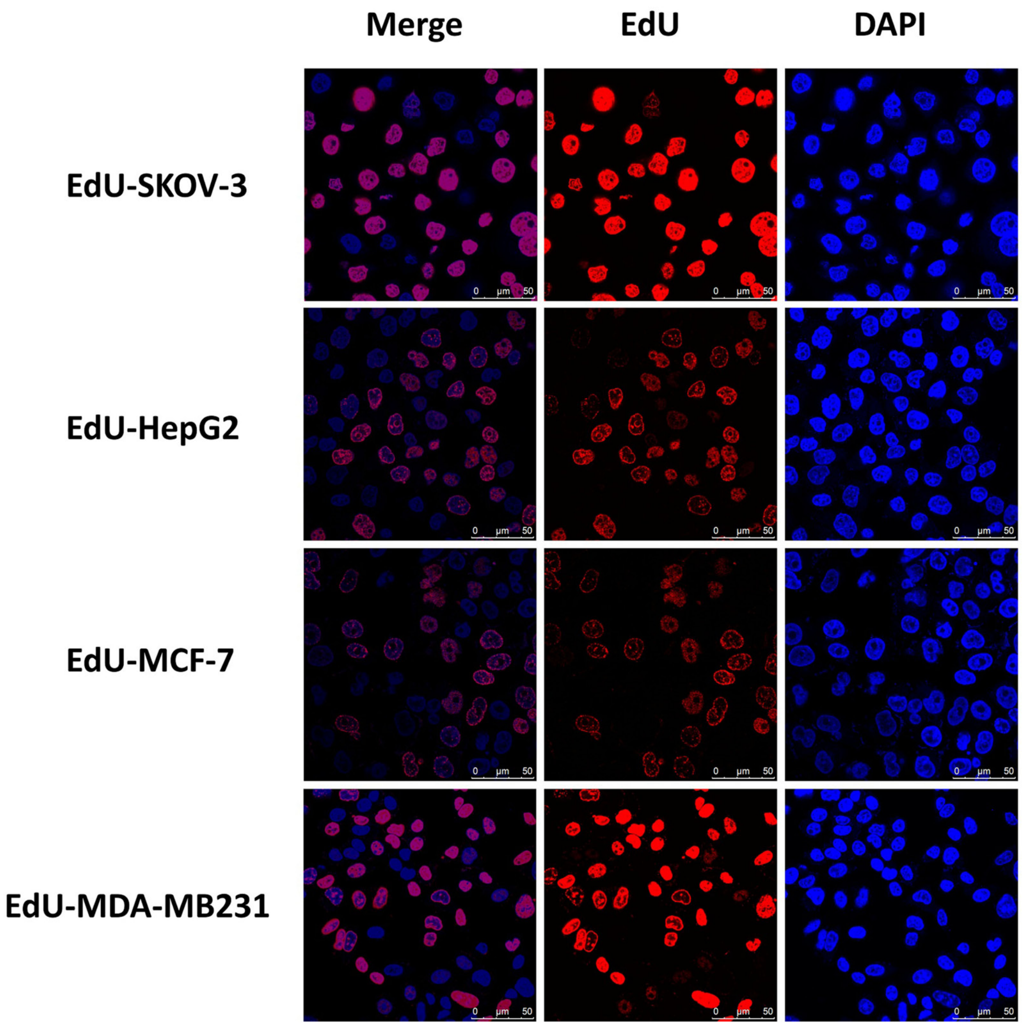


**Supplementary Figure 4: EdU labeling of MCF-7, HepG2, SKOV-3 and MDA-MB231 cell line DNA.** Detection of EdU shows that labeling was successful and that nearly all of the cells contain EdU (red) label. Nuclei were stained with DAPI (blue), with merged image on the left. Scale bar, 50µm.

A

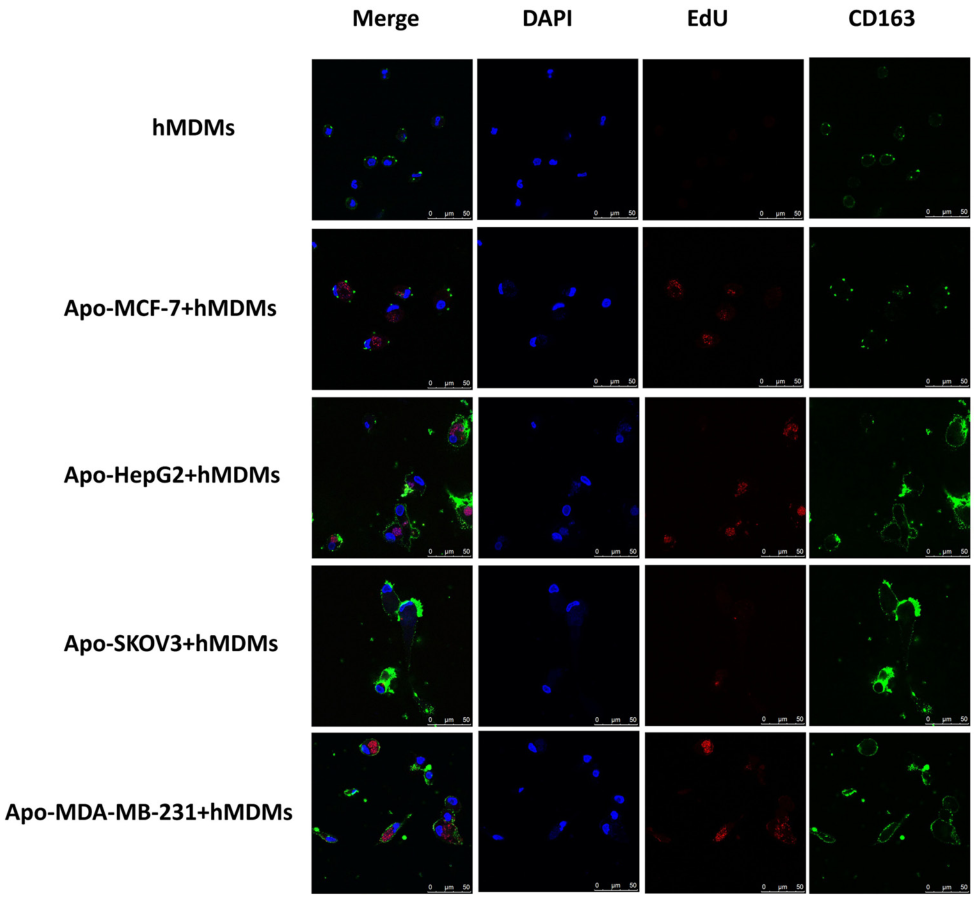


B

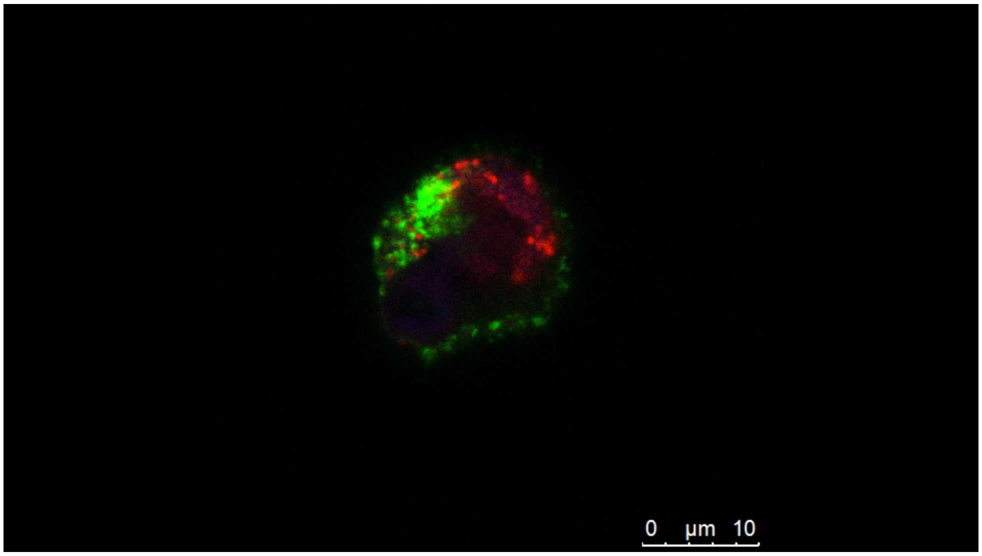


**Supplementary Figure 5: Confocal microscopy analysis and 3D reconstruction of CD163 and EdU expression in hMDMs co-cultured with apoptotic cells from different tumor cell lines.** Apoptotic tumor-derived DNA was labeled with EdU (red) and hMDMs were identified by CD163 (green) positivity. **(A)** Staining for nuclei (blue) apoptotic DNA (red) and hMDMs (green) with merged images shown to the left for hMDMs and hMDMs co-cultured with different types of apoptotic cancer cell lines. Presence of EDU in the cytoplasm of hMDMs indicates that hMDMs engulfed debris from apoptotic tumor cells. Scale bar, 50µm. **(B)** Dynamic map of the 3D reconstruction of a cell from the co-culture of apoptotic MDA-MB231 cells with hMDMs shown in figure 5A.

See Supplementary Video 1

**Supplementary Table 1: PCR primers for assessing expression of tumor and monocyte/macrophage markers by qRT-PCR**

| Tumor marker | | Monocyte/macrophage | |
|---|---|---|---|
| **Name** | **Sequence** | **Name** | **Sequence** |
| EpCAM | Forward<br>5'-TCTGAGCGAGTGAGAACCTAC -3' | CD163 | Forward<br>5' -TCTGGCTTGACAGCGTTTC -3' |
| | Reverse<br>5'-AGCACAACAATTCCAGCAAC -3' | | |
| HER2 | Forward<br>5'-AGGAGTGCGTGGAGGAAT-3' | | Reverse<br>5' -GTGTTTGTTGCCTGGATT -3' |
| | Reverse 5'-AGTGGGTGCAGTTGATGG-3' | | |
| MUC1 | Forward<br>5'-GCACCGACTACTACCAAGAG-3' | CD68 | Forward<br>5' -CGCAGCACAGTGGACATT -3' |
| | Reverse<br>5'-AAGGAAATGGCACATCACT-3' | | |
| EGFR | Forward<br>5'-GACCGTTTGGGAGTTGATG-3' | | Reverse<br>5' -AGGCCAAGAAGGATCAGG -3' |
| | Reverse<br>5'-GGGCGACTATCTGCGTCTA-3' | | |
| CEA | Forward<br>5'-CTGCTCACAGCCTCACTTCTAA- 3' | | |
| | Reverse<br>5'-GGGTAGCTTGTTGAGTTCCTATT- 3' | | |
